# Genome-Wide identification and salt stress-responsive expression dynamics of the HMGR gene family in *Ziziphus jujuba* var. *spinosa*

**DOI:** 10.1371/journal.pone.0330439

**Published:** 2025-08-20

**Authors:** Xiaohan Tang, Xiaojun Ma, Jun Cao, Xinhong Wang, Xuexiang Li, Xiaozhou Yang, Jing Shu

**Affiliations:** College of Forestry Engineering, Shandong Agriculture and Engineering University, Jinan, P R China; Arish university, Faculty of agricultural and environmental sciences, EGYPT

## Abstract

Terpenoids are critical components of plant environmental adaptation mechanisms. They also exhibit significant therapeutic potential in herbal medicine. 3-Hydroxy-3-methylglutaryl coenzyme A reductase (HMGR), a pivotal rate-limiting enzyme governing the initial stage of the mevalonate (MVA) pathway in triterpene saponin biosynthesis, remains uncharacterized in *Ziziphus jujuba* var. *spinosa*. Through genome-wide and molecular analysis, we systematically identified ZjHMGR isoforms and revealed differential tissue-specific expression patterns and significant salt stress-responsive regulation across identified isoforms. Our findings reveal three evolutionarily conserved ZjHMGR isoforms with a complete HMG-CoA reductase domain and closely related to *Populus trichocarpa*. Collinearity analysis revealed two collinear gene pairs, and purifying selection was identified as the primary evolutionary force acting on the *ZjHMGR* gene family. Cis-acting element analysis revealed that *ZjHMGR* gene family enriched MYB-related, TC-rich repeats, light- and hormone-responsive elements, suggesting transcriptional regulation by environmental stimuli and phytohormones. Spatiotemporal expression analysis via qRT-PCR revealed differential transcriptional patterns of *ZjHMGR* members, with pronounced upregulation under ABA, MeJA, and light induction. Saline stress disrupted the growth of wild jujube seedlings while activating *ZjHMGR* expression alongside other MVA pathway genes. Overexpression of *ZjHMGR* enhances salt stress resistance in *Arabidopsis thaliana*. This study lays the foundation for further investigations into the molecular mechanisms of the *ZjHMGR* gene family concerning saponin biosynthesis, phytohormone interactions, and salt tolerance in wild jujube.

## 1. Introduction

*Ziziphus jujuba* var. *spinosa* (Rhamnaceae) is a diploid progenitor of cultivated jujube indigenous to China. It represents a phylogenetically and economically significant species with dual medicinal-agronomic value [1]. This hardy perennial, colloquially termed “wild jujube,” serves as a critical genetic resource for arid-land agriculture due to its exceptional abiotic stress resilience, maintaining physiological homeostasis under saline conditions (5‰ NaCl) and nutrient-deficient soils [2]. Wild jujube serves as a key pioneer species for ecological restoration and soil conservation in harsh environments, evidenced by its capacity to thrive in marginal ecosystems, including saline-alkaline lands and barren mountainous regions [3]. The *Shennong Bencao Jing* (Divine Farmer’s Materia Medica, circa 200–300 CE), an ancient Chinese pharmacopeia, documents the phytotherapeutic efficacy of wild jujube in “regulating visceral homeostasis, enhancing metabolic regulation, and promoting longevity” [4]. Phytochemical profiling reveals therapeutic metabolites distributed across its leaves, fruits, flowers, and seeds, with particular pharmacological interest in its mature seeds (*suanzaoren*), a traditional Chinese medicinal material [4–6]. Modern pharmacological studies mechanistically link these traditional claims to the species’ bioactive triterpenoids and polysaccharides, which demonstrate multi-target regulatory effects on plant defense-immune networks, metabolism and oxidative stress pathways [1,2,4]. The species’ unique combination of stress tolerance traits and bioactive compound biosynthesis underscores its importance in sustainable crop development and natural product discovery.

Plants regulate complex adaptive mechanisms to counter dynamic environmental perturbations through evolutionarily conserved stress perception systems. These coordinated responses exhibit fine-tune in maintaining cellular osmotic equilibrium, redox homeostasis, and metabolic balance – fundamental requirements for environmental acclimatization [7–10]. The ability of plants to withstand extreme environmental challenges stems primarily from phylogenetically conserved specialized metabolic systems [11,12]. This evolutionary mechanism has given rise to three major groups of metabolites, phenolic derivatives including flavonoids, terpenoids including saponins, and alkaloids, which collectively mediate key physiological functions ranging from scavenging of reactive oxygen species (ROS) to phytohormone regulation, thereby maintaining cellar homeostasis in the face of abiotic and biotic stress [11,13,14]. Terpenoids represent the phylogenetically conserved secondary metabolites. They coordinate important plant-environment interactions through dual chemical-ecological functions, regulate interactions through organic compounds, and act as a regulatory link between phytohormone signaling [11].

The dual functionality of terpenoid metabolism in *Ziziphus jujuba* var. *spinosa* encompasses both medicinal biosynthesis [1,4] and stress adaptogenesis [15–17]. Structural elucidation identifies jujuboside saponins – dammarane triterpenoids isoprenylated as pharmacologically active analogs to panaxosides [4,5]. Simultaneously, terpenoid-based hormonal regulators, notably abscisic acid (ABA) and gibberellin (GA3) mediate cross-talk between phytohormone signaling, reinforcing membrane integrity through sterol-lipid integration, and direct ROS scavenging through conjugated isoprene units, thereby coordinating growth-defense tradeoffs under stressful constraints [9,18–20]. Evolutionary analyses reveal terpenoid indispensable role in plant stress resilience, particularly through compartmentalized biosynthesis during abiotic/biotic stressors. Crucially, terpenoid diversification patterns correlate with stressful adaptation, demonstrating selective pressure maintenance of their biosynthetic gene clusters [11]. The metabolic crosstalk between terpenoid pathways and phenolic antioxidant networks establishes a robust defense continuum, positioning these compounds as fundamental components of plant adaptive arsenals.

Plant terpenoid biosynthesis operates through two compartmentalized pathways, the cytosolic mevalonate (MVA) route and plastidial methylerythritol phosphate (MEP) system [21]. 3-Hydroxy-3-methylglutaryl-CoA reductase (HMGR), the principal flux-controlling enzyme in MVA metabolism, catalyzes the NADPH-dependent irreversible reduction of HMG-CoA to mevalonate [22,23]. This committed step governs carbon partitioning into sesquiterpenoids, phytosterols, and bioactive triterpenes through substrate channeling mechanisms, while spatial segregation of MEP-derived isoprenoids prevents metabolic cross-talk between organelles [11,24,25].

HMGR multigene family exhibits evolutionary diversification across plant taxa, with molecular characterization extending from model *Arabidopsis thaliana* [26,27], to pharmacologically significant species (*Panax ginseng* [28]*, Salvia miltiorrhiza* [29]*, Ginkgo biloba* [30], *Lithospermum erythrorhizon* [23] and *Taxus* [24]) and agronomically vital crops (*Glycine max* [31] and *Vitis vinifera* [32]). Functional genomics reveal HMGR’s dual regulatory capacity, transgenic overexpression enhances terpenoid biosynthetic flux while conferring growth-defense tradeoff advantages [22,28,31,33,34]. Photoregulation mechanisms were demonstrated in *Solanum tuberosum*, where HMGR activity undergoes light-dependent post-translational modulation – dark-induced suppression and phytochrome-mediated recovery [35]. Cross-species analyses in *Malus domestica* domestica illustrate HMGR’s pleiotropic effects, *MdHMGR5* ectopic expression coordinately upregulates MVA/MEP pathway genes, enhances ROS scavenging capacity, and promotes stress-adaptive morphogenesis [34]. *Taraxacum kok-saghyz* genome analysis identified HMGR promoters enriched in cis-regulatory elements (containing methyl jasmonate (MeJA)/ethylene-responsive motifs), with hormonal induction triggering coordinated metabolic reprogramming – HMGR upregulation paralleled by osmoprotectant accumulation and photosynthetic adaptation [22].

The halophytic adaptation of *Ziziphus jujuba* var. *spinosa*, a halophytic species with dual medicinal-edible value, manifested through its oligotrophic resilience and xerophytic endurance, remains mechanistically enigmatic despite extensive characterization of its triterpenoid biosynthesis since genome sequencing [36]. The dynamic interplay between defensive terpenogenesis and environmental sensing in medicinal plants manifests through two regulatory axes: biotic stress-responsive synthesis of phytoanticipins and abiotic stress-activated metabolic channeling via phytohormone-ROS signaling hubs. Cross-kingdom RNAi studies reveal UVR8-dependent MAPK cascades transduce light stress into monoterpene synthase activation [37], while drought/salinity co-opt ABA-JAZ modules may to redirect MEP/MVA flux toward stress-protective triterpenoids [11,18]. Notably, the isoprenoid-derived phytohormone ABA orchestrates multilevel stress adaptation via guard cell dynamics regulation, ionic equilibrium maintenance, and stress-responsive transcriptome reprogramming [9].

This study focus on a comprehensive investigation of the *ZjHMGR* gene family, a potential genetic locus underlying the exceptional stress resilience of *Ziziphus jujuba* var. *spinosa*. Our genome-wide characterization systematically delineates chromosomal distribution patterns, protein structural dynamics and physicochemical profiles, evolutionary conservation of functional motifs/domains, stress-responsive cis-regulatory architectures in promoter regions, phylogenetic divergence across angiosperm taxa, and tissue-specific spatiotemporal expression signatures. Functional interrogation via heterologous overexpression models analyzed specific functional stratification, with *ZjHMGR1/2/3* exhibiting distinct salt tolerance modulation capacities. Through integrated multi-omics analysis, we have elucidated a tripartite regulatory cascade encompassing upstream transcriptional orchestration, dynamic expression fine-tuning, and downstream metabolic network recalibration. The mechanistic dissection reveals ZjHMGRs’ multifunctionality in abiotic stress perception and signaling transduction, with particular emphasis on their pleiotropic regulation through isoprenoid biosynthesis pathways. Our findings establish novel regulatory modules that bridge genetic architecture with metabolic reprogramming under stress conditions. These discoveries providean evolutionary framework for extremophyte genetic resource exploitation and synthetic biology blueprints for salinity-resilient crop engineering. By resolving the ZjHMGR-mediated stress adaptation paradigm, this work advances both fundamental understanding of plant-environment interactions and translational applications in sustainable agriculture.

## 2. Materials and methods

### 2.1. Genome-wide identification of *HMGR* genes in *Ziziphus jujuba* var. *spinosa*

Based on the published whole genome data of *Ziziphus jujuba* var. *spinosa* [36], the genome sequence and annotation information of wild jujube were downloaded from NCBI to build a local BLAST database. Using the *Arabidopsis* HMGR protein sequence as the query sequence, local BLASTp was performed to obtain the *HMGR* gene family members of *Ziziphus jujuba* var. *spinosa* using BioEdit software. Then, the Pfam database (https://www.ebi.ac.uk/jdispatcher/pfa/pfamscan) and NCBI-CDD database (https://www.ncbi.nlm.nih.gov/Structure/cdd/wrpsb.cgi) were used to identify the HMGR family members of *Ziziphus jujuba* var. *spinosa*. Chromosomal localisation and gene structure analysis of *ZjHMGRs* were performed using Tbtools software [38].

### 2.2. Physicochemical properties, multiple sequence alignments, motif, conserved domain analysis and protein structure prediction

The physicochemical properties of the ZjHMGR proteins, including their molecular weight and isoelectric point, were analysed by the Protparam tool, which is available online via Expasy (v2023.1) [39] (https://web.expasy.org/protparam/).The conserved domains and conserved motifs of the three ZjHMGR proteins were analyzed via the SMART DOMAIN [40] (https://smart.embl.de/smart/set_mode.cgi?NORMAL=1) and MEME suit [41] (http://memesuite.org/tools/meme), and the maximum number of motifs was 10. Visualization of conserved motifs and structural domains by Tbtools software. The transmembrane structural domains, signal peptide and subcellular localisation of ZjHMGR proteins were predicted and analysed via DeepTMHMM v2.0 [42] (https://dtu.biolib.com/DeepTMHMM), SignalP 6.0 [43] (https://services.healthtech.dtu.dk/services/SignalP-6.0/) and the Plant-mPLoc tool of Cell-PLoc 2.0 [44] (www.csbio.sjtu.edu.cn/bioinf/plant-multi/), respectively. Multiple sequence alignments was analyzed by ESPript 3.0 [45] (https://espript.ibcp.fr/ESPript/ESPript/index.php). The secondary structure and 3D structure of the remaining proteins was predicted from the SOPMA (https://npsa-prabi.ibcp.fr/cgi-bin/npsa_automat.pl?page=npsa%20_sopma.html) and the AlphaFold3 [46] online website (https://deepmind.google/technologies/alphafold/alphafold-server/), separately.

### 2.3. Phylogenetic analysis, collinearity analysis and selective pressure discovery

The protein sequences of HMGRs in date palm and *Arabidopsis thaliana*, soybean, grape, apple, rice, mauve poplar, and corn were aligned using MUCLE in MEGA 11.0, and a phylogenetic tree was constructed with the maximum likelihood method with 1000 replications [47], to explore the evolutionary relationship of ZjHMGRs. The phylogenetic tree was beautified by the online website iTOL (https://itol.embl.de/). Intra- and inter-species collinearity analyses of the ZjHMGR gene were performed using the McScanX plug-in of the TBtools software. And the Ks values of collinear gene pairs were calculated and the divergence time was calculated with the formula T = Ks/2r × 10^−6^ (million years Mya), where Ks is one synonymous substitution at each locus and r is 1.5 × 10^−8^ per year at each locus for dicotyledons synonymous substitutions [48].

### 2.4. Cis-acting element analysis

Potential promoter sequences, 2000 bp upstream of the transcription start site of the ZjHMGR genes, were extracted by TBtools, analyzed for promoter region cis-acting element prediction by PlantCARE database [49] (http://bioinformatics.psb.ugent.be/webtools/plantcare/html/), and subsequently visualized by TBtools.

### 2.5. *Ziziphus jujuba* var. *spinosa* growth and sample collection

The seeds (ZH#1 and ZH#2) of *Ziziphus jujuba* var. *spinosa* were obtained from Zanhuang Country, Hebei, China. *Ziziphus jujuba* var. *spinosa* (wild jujube) is widely distributed and cultivated in China. The ZH#1 and ZH#2 germplasm used are not listed on any protected plant or animal conservation lists. Unless otherwise specified, the ZH#1 germplasm served as the primary plant material in this study. Seeds were soaked at 30 ^◦^C for 2–3 days before germination, and plants were grown under continuous white light at 23 ± 2 ^◦^C in soil. Roots, stems, leaves and flowers were collected from five -year-old plants grown in a greenhouse. Four- to six-leaf stage seedings grown at hydroponic tank were selected for treatment analysis. Seedings were treated with NaCl/Na_2_SO4/NaHCO_3_/Na_2_CO_3_ 1:1:1:1 M ratio for 0–4 d to simulate salinity stress. For hormone treatment, seedings were irrigated with 100 μmol/L ABA or 1 mmol/ MeJA, and samples were collected at different periods of 0 h, 6 h, 12 h, 24 h, respectively. Following a regular growth stage, the seedlings were transferred to darkness for 24 h and then returned to light for a further 0–24 h recovery, to analyze effects of light on *ZjHMGR* expression. All samples were quick-frozen in liquid nitrogen and stored at −80 ◦C.

### 2.6. Physiological indicators and antioxidant enzyme assays

Four to six leaf stage seedings grown at hydroponic tank were treated with NaCl/Na_2_SO4/NaHCO_3_/Na_2_CO_3_ 1:1:1:1 M ratio for 0−8 d to simulate salinity stress. Quantification of proline, soluble sugar, chlorophyll and antioxidant enzyme was conducted on ~50 mg of fresh sample. The chlorophyll contents of seedings were determined spectrophotometrically from the absorbance at 663 nm and 649 nm following standard extraction. The contents of proline and soluble sugar in samples was determined in accordance with the assay kits (Nanjing Jiancheng Bioengineering Institute, cat#A107-1–1 and cat#A145-1–1, China).CAT, SOD, and POD activity of seedings were analyzed in accordance with the assay kits (Nanjing Jiancheng Bioengineering Institute, cat# A007-1–1, cat# A001-1–2, and cat# A084-3–1). All spectrophotometric analyses were conducted on a BMG SPECTRO star omega microplate reader (BMG Labtech, Germany). All data were obtained from at least three biological replicates.

### 2.7. RNA extraction and qRT-PCR analysis

Total RNA Extraction Reagent (Vazyme, R401), reverse transcription kit HiScript III RT SuperMix for qRTPCR (+gDNA wiper) (Vazyme, R323) and ChamQ Universal SYBR qRT-PCR Master Mix (Vazyme, Q711) were used for qRT-PCR following the manufacturer’s instructions. mRNA-sequencing was conducted using the high-throughput Genomics Core facility at Novomagic (www.novogene.cn). Gene expression was normalized to that of *AtACTIN8* or *ZjACTIN7* and then the expression level in the different timepoint was determined relative to the level in the control plants. qRT-PCR analysis was performed with 3 independent biological replicates, and the data were analysed using the 2^–ΔΔ^CT method [50] with mean *± *standard deviation (SD).Statistical differences were shown by different letters according to One-Way ANOVA followed by Tukey’s HSD test, *P < 0.05. The specific primers used for this paper are listed in Supplementary S2 Table.

### 2.8. Plasmid construction and plant transformation

To construct gene fusions with *pROKII-X-eGFP*, the coding sequences (removed the stop codon) of *ZjHMGRs* were each individually amplified through PCR, cloned, and inserted into the corresponding vectors using a ClonExpress II One Step Cloning Kit (Vazyme, C112). The construct *pROKII-ZjHMGR1/2/3-eGFP* was transformed into Col-0 (*Arabidopsis thaliana*) by the floral dip method. Based on the selection with antibiotics (using 50 milligrams per liter of kanamycin), a T1 generation transgenic population was cultivated. Subsequent generations were screened until complete homozygosity was achieved. The transformed individuals were then used for subsequent functional studies. After vernalizing at 4 °C for 24 h, the transgenic lines of *A. thaliana* grew in 1/2MS medium at 21°C under 16-h-light/8-h-dark conditions. Transgenic homozygous lines were utilized to detect biomass and observe root and shoot growth. The main root’s length was measured by ImageJ. The fresh weight and dry weight was analyzed by using fresh seedings of 8-day-old *A. thaliana*. The primer pairs are listed in Supplementary S3 Table, and all the generated constructs were confirmed by sequencing.

## 3. Results

### 3.1. Identification of HMGR Family Members in *Ziziphus jujuba* var. *spinosa*

The *Ziziphus jujuba* var. *spinosa* genome was systematically interrogated through homology-based BLASTp analysis employing the *Arabidopsis thaliana* HMGR protein sequence (AT2G17370) as the query. A total of three *HMGRs* were identified in the *Ziziphus jujuba* var. *spinosa* genome, and the members were named *ZjHMGR1* ~ *ZjHMGR3* according to the order of localization in the chromosome.

Chromosome distribution mapping revealed the ZjHMGR paralogs reside on chromosomes 6, 8, and 9, with ZjHMGR1-ZjHMGR3 names assigned according to their linear genomic arrangement (S1 Fig in S1 File). Comparative analysis of gene architecture demonstrated substantial structural diversities among family members, with gene lengths ranging from 2,914 bp to 4,012 bp, corresponding to coding sequence (CDS) lengths of 1,710−1,812 bp (Table 1). Strikingly, ZjHMGR3 exhibits a paradoxical genomic architecture – despite possessing the most compact genomic, it contains the longest CDS to code protein (Table 1). This intriguing structural feature suggests ZjHMGR3 may have evolved distinct regulatory mechanisms, potentially through intron loss or alternative splicing patterns not observed in ZjHMGR1 or ZjHMGR2.

**Table 1:** The detail information of *HMGR* gene family members in *Ziziphus jujuba* var. *spinosa.*

Gene name	Gene ID	Chromosomal localization	Gene length/bp	Location	CDS length/bp
ZjHMGR1	ZspiChr6G00030740.1	Chr6	3864	782618-786481	1710
ZjHMGR2	ZspiChr8G00149430.1	Chr8	4012	8050134-8054145	1758
ZjHMGR3	ZspiChr9G00131940.1	Chr9	2914	19404627-19407540	1812

Comprehensive biophysical characterization of ZjHMGR isoforms was performed through computational physiochemical profiling using the Expasy ProtParam platform (v2023.1). The analysis revealed conserved polypeptide lengths (569–603 residues) across family members, corresponding to predicted molecular masses of 60.88–64.68 kDa. Intriguingly, ZjHMGR1 (pI 7.59) and ZjHMGR2 (pI 7.17) exhibited alkaline isoelectric points with elevated instability indices (42.6 and 41.75 respectively), classifying them as intrinsically disordered proteins through threshold >40. In contrast, ZjHMGR3 demonstrated divergent biophysical behavior with a mildly acidic pI (6) and enhanced thermodynamic stability (instability index 38.25), suggesting potential structural stabilization through distinct hydrophobic moment analysis. Subcellular compartmentalization predictions unanimously localized all three paralogs to the endoplasmic reticulum membrane system, corroborated by conserved dual transmembrane helices (TMHMM v2.0) – a structural hallmark of sterol biosynthetic enzymes (Table 2). This membrane-anchored configuration aligns with HMGR’s established role in mevalonate pathway channeling, though ZjHMGR3’s atypical C-terminal amphipathic helix implies potential isoform-specific regulatory interactions.

**Table 2:** The information of physicochemical properties of ZjHMGRs family proteins.

Gene name	Protein Length/aa	Molecular Weight/kDa	pI^1^	Instability Index	Predicted Location(s)	Number of TMHs^3^
ZjHMGR1	569	60.88	7.59	42.6	ER^2^	2
ZjHMGR2	585	63.16	7.17	41.75	ER	2
ZjHMGR3	603	64.68	6	38.25	ER	2

^1^ PI: Isoelectric point. ^2^ ER: Endoplasmic reticulum. ^3^ TMH: Transmembrane helices.

### 3.2. Variation across the ZjHMGR Family in terms of domain, motif composition, and conserved structure

**Fig 1:**
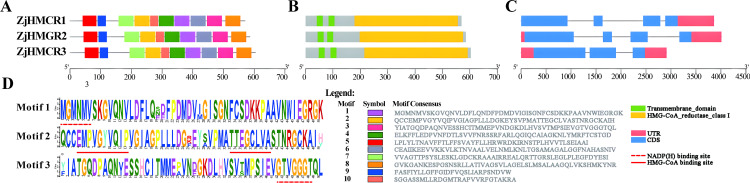
Conserved domain, motif analysis, and gene structure of the *ZjHMGR* family members in *Ziziphus jujuba* var. *spinosa.* (A) The top ten conserved motifs distribution of ZjHMGR proteins; the color boxes represent different conserved motifs, as shown in the scheme on the lower right side of the figure. (B) Predicted the distribution of conserved domains in ZjHMGR proteins. The green and yellow boxes represent the transmembrane domain and HMG-CoA reductase class I domains, respectively. (C) The exon–intron structures of *ZjHMGR* genes. The utilisation of pink boxes and light blue boxes to denote UTR and CDS, respectively, alongside the representation of introns by thin grey lines, serves to facilitate comprehension of the intricacies of the gene structure. (D) Three conserved motif logos including HMG-CoA binding sites and NADP(H) binding sites. Amino acids are represented by one-letter codes and presented in different colors.

This computational screening revealed three putative ZjHMGR homologs (ZjHMGR1–3) that were subsequently subjected to rigorous domain architecture validation using both Pfam (v35.0) and NCBI’s Conserved Domain Database (CDD v3.20). All candidate proteins exhibited the characteristic sterol-sensing domain (SSD, PF00368) and catalytic HMG-CoA reductase domain (cl00205), confirming their classification as bona fide members of the plant HMGR class I family (cd00643), as determined by iterative profile-profile alignment. To elucidate structure-function relationships within the ZjHMGR proteoforms, we performed integrative structural analyses combining multiple sequence alignment, secondary structure prediction, and 3D modeling. Domain architecture interrogation revealed all isoforms possess the canonical plant HMGR organization – ten evolutionarily conserved motifs and the HMG-CoA reductase І catalytic core (Fig 1A and 1B). Phylogenetic conservation of gene architecture is frequently observed among paralogous enzymes, yet comparative exon-intron mapping uncovered atypical structural compression in ZjHMGR3. While ZjHMGR1 (4 exons; 3,864 bp) and ZjHMGR2 (4 exons; 4,012 bp) maintain conserved splicing patterns, ZjHMGR3 (3 exons; 2,914 bp) achieves comparable coding capacity (1,812 bp CDS) through intronic optimization – a phenomenon potentially arising from retrotransposon-mediated intron loss (Fig 1C). Critical functional dissection of conserved motifs identified three catalytic hotspots: Motif 1 (NADP(H)-binding fingerprint), Motif 2 (dual HMG-CoA recognition pockets), and Motif 3 (NADP^+^ coordination) (Fig 1D).

**Fig 2:**
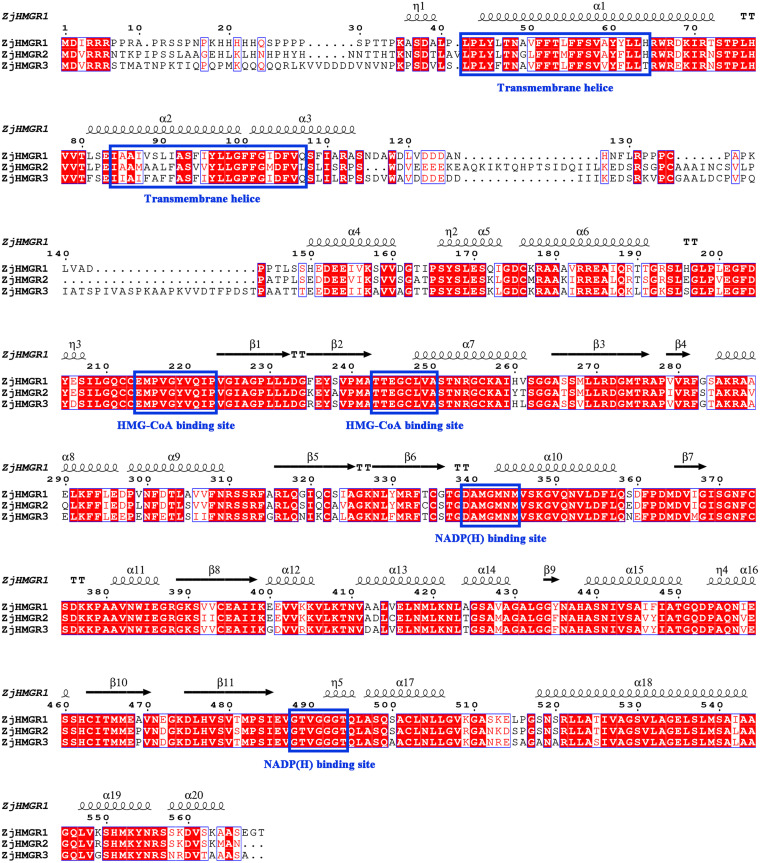
Multiple sequence alignment of the ZjHMGR proteins. The secondary structural elements predicted with ZjHMGR1 are shown above. Red box, white character represents strict identity; red character represents similarity in a group; blue frame represents similarity across groups. The η symbol refers to 3_10_-helixs; α-helixs and 3_10_-helixs are displayed as solenoids with numbers, respectively. β-strands are displayed as black arrows; strict β-turns are denoted as TT letters. The orange line represents three different transmembrane helices. The blue boxes with letters represent two transmembrane helices, two HMG-CoA binding sites and two NADP(H) binding sites, respectively.

Phylogenetic sequence analysis revealed pronounced evolutionary divergence between ZjHMGR paralogs, with the two N-terminal transmembrane domains at residues 43−64 and residues 84−107 exhibiting similarities (Fig 2). In contrast, the C-terminal catalytic domain demonstrated exceptional conservation, preserving two canonical HMG-CoA binding pockets and dual NADP(H)-interacting sites (Fig 1D and Fig 2), suggesting strong purifying selection pressure to maintain catalytic fidelity. Multiple sequence alignment using DeepTMHMM corroborated dual transmembrane helices in all isoforms, while SignalP-6.0 excluded secretory pathway involvement (Fig 2 and Table 2). These computational findings, combined with endoplasmic reticulum (ER) membrane localization predictions, support HMGR membrane protein configuration anchored to the ER membrane by two transmembrane movements (Fig 2 and Table 2).

The secondary structure predicted via ZjHMGR1 was shown above the multiple sequence alignments, with twenty α-helices, eleven β-strands, six strict β-turns, and five 3_10_-helixs (Fig 2). The first 3_10_-helixs is more divergent in the three ZjHMGR proteins. And the third 3_10_-helixs in ZjHMGR3 is different from other two ZjHMGR proteins, but the second, the fourth and the fifth 3_10_-helixs are the same in all three ZjHMGR proteins (Fig 2). The prediction of the secondary structure of the ZjHMGR protein family (Table 3) via SOPMA revealed that all ZjHMGR family members consisted of α-helices, β-extended strands, β-turns, and irregular curls. Among them, α-helices (41.96%−46.05%) had the highest percentage and followed by random curl structures (36.03%−38.47%) indicating that the secondary structure of ZjHMGR was mainly composed of α-helices and random curl and exercised the main functions, highlighting their potential functional significance in catalytic mechanisms and substrate interactions.

**Table 3:** The secondary structure of ZjHMGR family proteins.

Gene name	α-helix	β-strands	β-turn	Random coil
ZjHMGR1	45.34%	13.71%	4.92%	36.03%
ZjHMGR2	46.05%	12.20%	4.81%	36.94%
ZjHMGR3	41.96%	14.59%	4.98%	38.47%

**Fig 3:**
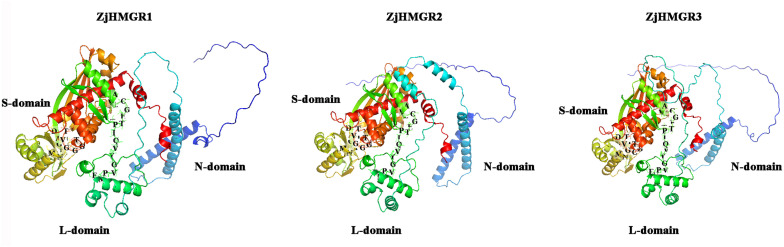
Predicted tertiary structures of ZjHMGR isoforms. ZjHMGR had three domains. N-domain (blue; small helical structure with N-terminal site, membrane anchoring), L-domain (green; ZjHMGR proteins structural center assembly with large helical structure containing HMG-COA and NADP(H) binding motifs), and S-domain (orange; small helical structures with NADP(H) binding motif, auxiliary NADPH coordination). Structural variations in the L-domain (e.g., loop topology, surface charge distribution) may facilitate isoform-specific regulatory mechanisms or protein-protein interactions under stress. Models generated via AlphaFold. Image coloured by rainbow N to C terminus.

The tertiary structure-function relationship of proteins constitutes a cornerstone of regulatory mechanism. Based on the highly conserved protein sequences of the ZjHMGR family members, we constructed and mapped 3D protein structural models to distinguish structural divergence patterns across this enzyme family through AlphaFold3 to facilitate a better understanding of ZjHMGR family protein functions. Comparative analysis of predicted models demonstrated striking structural conservation among ZjHMGR isoforms, with all members adopting a tripartite domain organization: a N-domain with small helical structure of N-terminal site mediating membrane association, a L-domain housing large helical structure containing conserved HMG-COA and NADP(H) binding motifs, and a small helical structure with NADP(H) binding motif on catalytic S-domain (Fig 3). ZjHMGR are highly conserved, extending from primary sequences to tertiary structural features, during the evolutionary process and may perform analogous functions, suggesting strong purifying selection acting on ZjHMGR paralogs during Ziziphus jujuba speciation. These structural conservation features of ZjHMGR may underpin functional redundancy in isoprenoid biosynthesis, potentially serving as evolutionary safeguards to maintain metabolic flux homeostasis.

### 3.3. Phylogenetic relationships, collinearity analysis and selective pressure discovery of hmgr family members in *Ziziphus jujuba* var. *spinosa*

**Fig 4:**
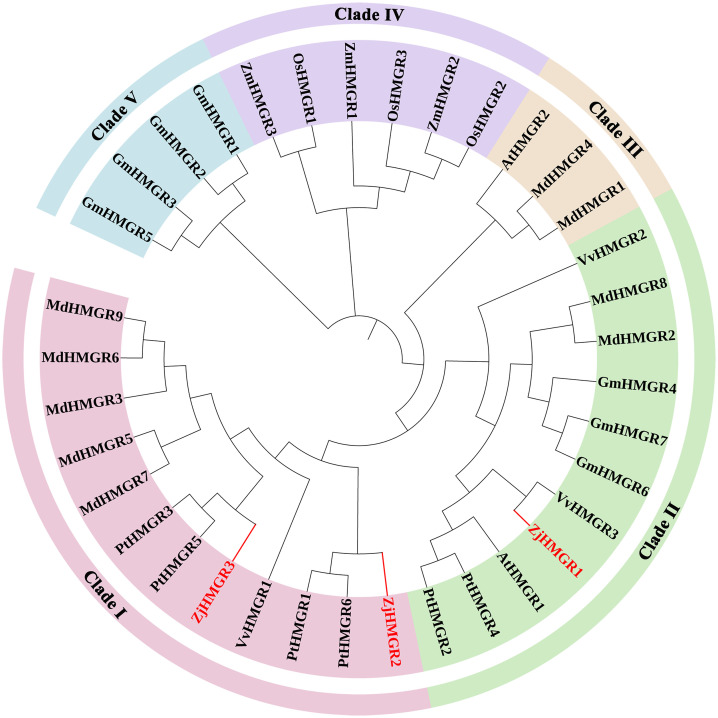
Evolution analysis of *HMGR* genes in *Ziziphus jujuba* var. *spinosa* (Zj), *Arabidopsis thaliana* (At), *Glycine max* (Gm), *Oryza sativa* (Os), *Malus domestica* (Md), *Populus trichocarpa* (Pt), *Vitis vinifera* (Vv), and *Zea mays* (Zm). The tree was constructed with MEGA 11.0 using the maximum likelihood (ML) method with 1000 bootstrap replications. Detailed Gene ID and protein sequences in Supplementary S4 Table.

To elucidate the evolutionary relationships of HMGR in *Ziziphus jujuba* var. *spinosa* relative to other plants, we performed maximum likelihood (ML) phylogenetic analysis using protein sequences from eight representative species. The dataset comprised three ZjHMGRs along with orthologs from *Arabidopsis thaliana* (2 AtHMGRs), *Glycine max* (7 GmHMGRs), *Malus domestica* (9 MdHMGRs), *Oryza sativa* (3 OsHMGRs), *Populus trichocarpa* (6 PtHMGRs), *Vitis vinifera* (3 VvHMGRs), and *Zea mays* (3 ZmHMGRs), totaling 36 HMGR sequences encompassing both monocotyledonous and dicotyledonous species. HMGR proteins were classed into five well-supported clades (I-V) with significant bootstrap values (Fig 4 and S4 Table). Notably, ZjHMGR1 exhibited closest phylogenetic affinity to VvHMGR3 within clade II, demonstrating the strongest sequence identity. The remaining isoforms ZjHMGR2 and ZjHMGR3 were clustered in clade I with PtHMGR homologs, showing highly conservation. This grouping pattern suggests conserved evolutionary approaches between ZjHMGRs and those from woody perennials in *Populus trichocarpa* and *Vitis vinifera*.

**Fig 5:**
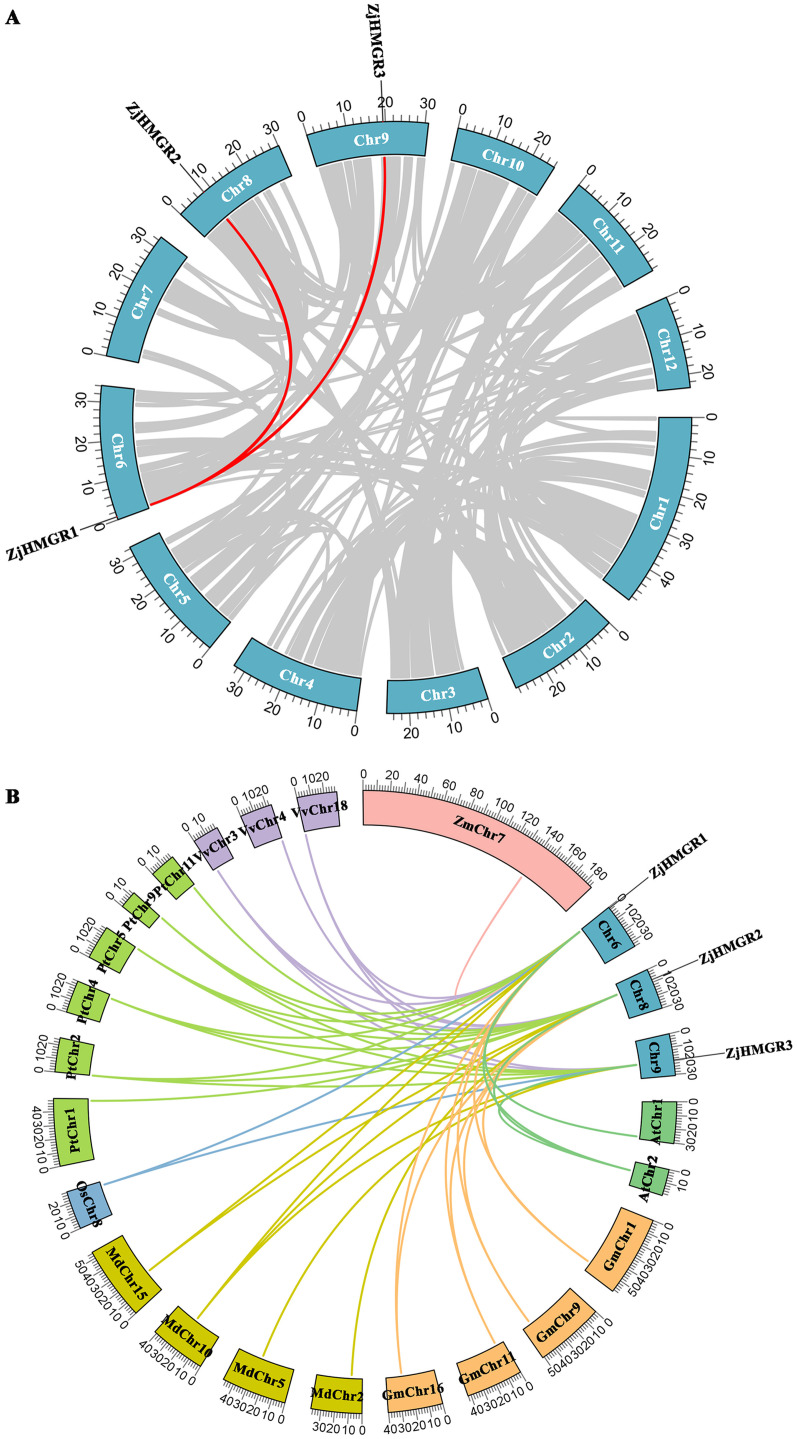
Collinearity Analysis of *ZjHMGR* genes. (A) Chromosomal distribution and intraspecific covariance analysis of the *ZjHMGR* genes. Red lines connected to blue boxes represent *ZjHMGR* gene pairs, and gray lines represent homologous modules in the background of the wild jujube genome. (B) Covariance analysis of HMGR across *Ziziphus jujuba* var. *spinosa* (Zj), *Arabidopsis thaliana* (At), *Glycine max* (Gm), *Oryza sativa* (Os), *Malus domestica* (Md), *Populus trichocarpa* (Pt), *Vitis vinifera* (Vv), and *Zea mays* (Zm) chromosomes. The colored lines highlight the collinear blocks in the genomes of *Ziziphus jujuba* var. *spinosa* and the other seven plant species. Various plant species were marked as boxes with different colors.

Gene duplication mechanisms, fundamental drivers of genomic and gene family diversification, were systematically investigated in the ZjHMGR family. Phylogenomic reconstruction revealed that all ZjHMGR paralogs originated through either whole-genome duplication (WGD) or segmental duplication events. Two paralog pairs (*ZjHMGR1–ZjHMGR2* and *ZjHMGR1–ZjHMGR3*) exhibit significant collinearity, localized to chromosomes 6, 8, and 9 (Fig 5A). This spatial separation confirms their origin via segmental duplication rather than tandem duplication. To quantify evolutionary constraints, we calculated pairwise non-synonymous (Ka) and synonymous (Ks) substitution rates, deriving purifying selection pressure indices (Ka/Ks ratios). Both paralog pairs (ZjHMGR1–ZjHMGR2 and ZjHMGR1–ZjHMGR3) exhibit Ka/Ks ratios significantly below 1 (0.0565 and 0.0597, respectively; *P* < 0.001). This unequivocally indicates stringent purifying selection, preserving essential functional domains of HMGR across evolutionary time. Such conservation underscores HMGR’s indispensable role in the mevalonate pathway, where mutations in catalytic residues are likely deleterious (Table 4). The paralogs arose via segmental duplication at distinct timepoints. ZjHMGR1–ZjHMGR2 diverged 83.84 Mya (Late Cretaceous), ZjHMGR1–ZjHMGR3 diverged 82.96 Mya (Table 4). The ≈ 0.9-Mya lag between events aligns with independent genomic rearrangements, potentially coinciding with ancestral whole-genome duplication (WGD) episodes common in eudicots.. To elucidate the evolutionary dynamics of the HMGR gene family in *Ziziphus jujuba var. spinosa* (wild jujube), a comprehensive covariation analysis was conducted across phylogenetically diverse species, including *Arabidopsis thaliana*, *Glycine max*, *Malus domestica*, *Oryza sativa*, *Populus trichocarpa*, *Vitis vinifera*, and *Zea mays*. The analysis revealed 4, 8, 7, 2, 14, 7, and 1 collinear gene pairs between wild jujube and the aforementioned species, respectively (Fig 5B). Wild jujube shares the highest number of collinear *HMGR* pairs (14) with *P. trichocarpa,* underscoring evolutionary conservation of terpenoid pathway genes in perennial woody taxa. *ZjHMGR1* displays collinearity with monocots (*Oryza sativa*: 2 pairs; *Zea mays*: 1 pair), suggesting its emergence prior to monocot–eudicot divergence (~200 Mya). This positions *ZjHMGR1* as the ancestral lineage in the *ZjHMGR* family. Fewer collinear pairs with *Arabidopsis thaliana* (4) and *Glycine max* (8) highlight lineage-specific gene family expansions in eudicots. The ancient emergence and remarkable conservation of the *ZjHMGR* gene family may imply strong association with HMGR’s crucial role in MVA pathway regulation across divergent plant lineages.

**Table 4:** Detailed information on the Ka, Ks, Ka/Ks ratio and divergence-time in wild jujube.

Homologous gene pair	Ka	Ks	Ka/Ks	Divergence-Time/Mya^1^
ZjHMGR1-ZjHMGR2	0.142035	2.515159	0.056472	83.8386333
ZjHMGR1-ZjHMGR3	0.148599	2.488694	0.059709	82.9564667

Mya^1^: million years, T =Ks/2r ×10^-6^, r =1.5 × 10^−8^

### 3.4. Cis-acting element analysis of HMGR gene family in *Ziziphus jujuba* var. *spinosa*

**Fig 6:**
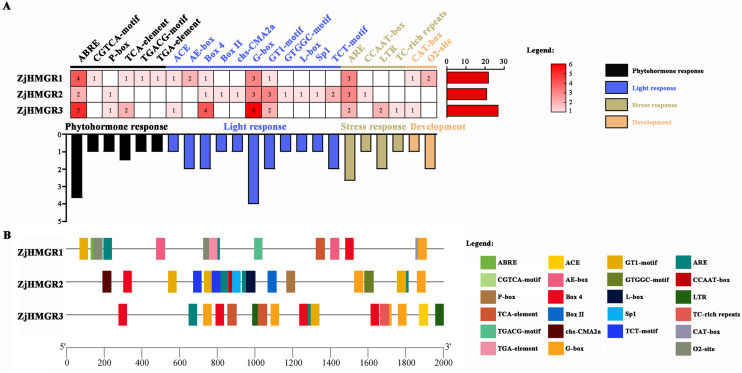
Prediction of cis-acting elements of promoters of the *ZjHMGR* gene family. PlantCARE was used to analyze the promoter sequences (2000 bp) of three *ZjHMGR* genes. (A) The numerical values denote the quantity of cis-acting elements of each type. (B)Vertical bars display the positional distribution of the cis-acting elements on the *ZjHMGR* promoters. In this legend, each color represented different cis-acting elements.

To investigate the functional potential of the ZjHMGR gene family in wild jujube, a systematic identification of *cis*-acting regulatory elements within its promoter regions was performed using the PlantCARE database. As illustrated in Fig 6, four major functional categories of *cis*-acting elements were identified after excluding ubiquitous TATA-boxes, CAAT-boxes, and one uncharacterized motif, including 35 light-responsive motifs (ACE, AE-box, Box 4, Box II, chs-CMA2a, G-box, GT1-motif, GTGGC-motif, L-box, Sp1, and TCT-motif), 19 hormone-responsive elements (abscisic acid-responsive element ABRE, growth hormone-responsive element TGA-element, gibberellin-responsive element P-box, methyl jasmonate-responsive elements CGTCA-motif and TGACG-motif, and salicylic acid-responsive element TCA-element), 12 stress-associated motifs (anaerobic induction element ARE, low-temperature responsiveness element LTR, defense/stress element TC-rich repeats, and MYBHv1 binding element CCAAT-box), and 4 growth/developmental regulators (meristem expression related CAT-box, and maize alkyd metabolism-regulatory element O2-site) (Fig 6A). Notably, three ZjHMGR isoforms exhibited conserved enrichment of key regulatory motifs, including light-responsive Box 4, G-box, and GT1-motif; abscisic acid-responsive ABRE; and anaerobic-inducible ARE (Fig 6B). These findings collectively suggest that ZjHMGR members may function as integrators of photomorphogenic signaling, abscisic acid-mediated stress adaptation, and hypoxia-responsive regulation. Furthermore, the co-occurrence of auxin (TGA-element), jasmonate (CGTCA/TGACG), and salicylic acid (TCA) response motifs across all three isoforms implies potential cross-talk between ZjHMGR activity and phytohormone signaling networks.

### 3.5. Tissue-specific expression of *ZjHMGR* and analysis of expression patterns upon different stresses and exogenous phytohormone treatments

**Fig 7:**
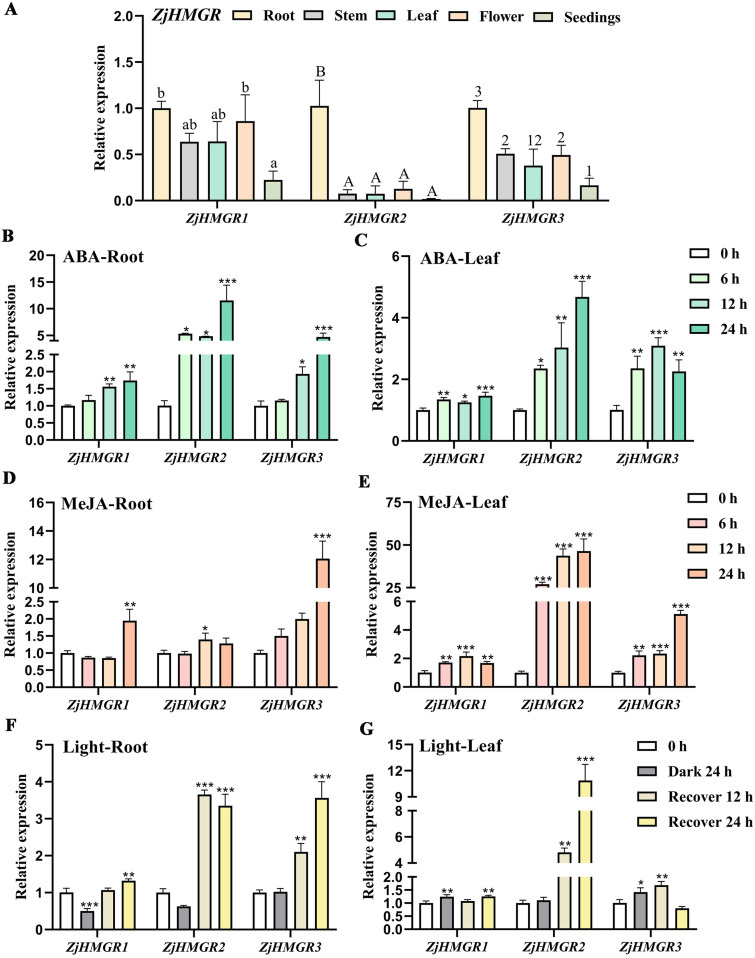
Tissue-specific expression of *ZjHMGR* and analysis of expression patterns upon different stresses and exogenous hormone treatments. (A) The transcription level of *ZjHMGR* in different tissues of 5-year-old plants and four- to six-leaf stage seedings assayed by qRT-PCR. (B–E) The transcription level of *ZjHMGR* in four- to six-leaf stage seedings treated with exogenous ABA or MeJA assayed by qRT-PCR. The levels in roots or leaves of seedlings in the absence or presence of 100 μM ABA or 1 mM MeJA for 0-24 h. (F-G) The transcription level of *ZjHMGR* in four- to six-leaf stage seedings protected from light for 24 h, and then recovered to light for 12 h or 24 h. The relative gene expression was normalized to the level of the control and was normalized to Zj*ACTIN7* expression. Data are from three biological replicates (± SD). Statistical differences were shown by different letters or asterisks according to One-Way ANOVA followed by Tukey’s HSD test, *P < 0.05, **P < 0.01, ***P < 0.01.

To elucidate the functional roles of *ZjHMGR* gene family members across various tissues and developmental phases in wild jujube, we conducted a comprehensive expression profiling analysis through qRT-PCR. The investigation revealed distinct tissue-specific expression profiles and significant inter-member transcriptional variations among the three ZjHMGR paralogs in root, stem, leaf, floral, and seedling tissues. Notably, comparative expression analysis demonstrated substantial transcriptional divergence between ZjHMGR isoforms. While ZjHMGR2 maintained relatively basal expression levels across all examined tissues, ZjHMGR1 and ZjHMGR3 exhibited comparable transcriptional abundance with significantly elevated expression intensities (Fig 7A). A particularly striking expression pattern emerged in root tissues, where all three ZjHMGR homologs displayed markedly higher transcript accumulation compared to other organ systems, suggesting potential isoform-specific functional specialization in belowground metabolic processes. Given the presence of phytohormone-responsive (ABA/MeJA) and light-regulatory *cis*-elements in all ZjHMGR paralog promoters, we systematically investigated the transcriptional dynamics of these isoforms under exogenous abscisic acid (ABA), methyl jasmonate (MeJA), and photoperiod treatments in *wild jujube.* To account for tissue-specific expression patterns observed in previous analyses (Fig 7A), experimental materials were standardized using wild jujube seedlings at the 4–6 true leaf developmental stage, with parallel expression profiling conducted in root and leaf tissues through qRT-PCR. Notably, ABA induction triggered coordinated transcriptional responses across the ZjHMGR family members, though with distinct pattern variations. All three paralogs exhibited significant upregulation following ABA treatment, with ZjHMGR2 demonstrating both the most rapid response and the highest magnitude induction. This differential regulation pattern suggests potential functional diversification among paralogs in ABA-mediated stress adaptation mechanisms. Quantitative profiling revealed temporally coordinated induction patterns of ZjHMGR1 in both root and leaf tissues during early ABA exposure (Fig 7B and 7C). In contrast, ZjHMGR3 displayed tissue-specific regulatory divergence, root tissues maintained progressive transcriptional activation throughout the treatment period, whereas foliar expression peaked at 12 h followed by a significant attenuation, suggesting potential feedback inhibition or post-transcriptional regulation in leaves. MeJA responsiveness uncovered striking disparities between hormone signaling pathways in ZjHMGR members. Root tissues exhibited delayed MeJA responsiveness relative to ABA stimulation, with ZjHMGR2 demonstrating primary response activation at 12 h, while ZjHMGR1 and ZjHMGR3 showed deferred upregulation until 24 h (Fig 7D and 7E). Notably, ZjHMGR3 achieved maximal induction amplitude despite its delayed activation, implicating isoform-specific roles in sustained JA signaling. This delayed responsiveness in ZjHMGR3 may be correlated with promoter architecture of higher abundance of ABA-responsive *cis*-elements compared to MeJA-responsive elements across ZjHMGR promoters. The differential response pattern with rapid ABA and delayed MeJA coupled with fine modulation among ZjHMGR paralogs suggest functional diversification in ABA-JA hormone signaling integration.

Based on the pronounced enrichment of light-responsive *cis*-elements in *ZjHMGR* promoters, we detected expression pattern of this gene family through controlled dark adaptation (24 h) and subsequent light recovery assays. In roots, dark-induced transcriptional attenuation manifested differentially across ZjHMGR paralogs, with ZjHMGR1 and ZjHMGR2 exhibiting significant downregulation, while ZjHMGR3 maintained basal expression levels under darkness (Fig 7F and 7G). This specific sensitivity to light suggests functional stratification, where ZjHMGR1/2 may act as light sensors in subterranean organs. All three paralogs showed light upregulation during dark treatment in leaves. This paradoxical induction correlates with the presence of chloroplasts or ROS bursts. Light restoration elicited transcriptional activation across all tissues, with ZjHMGR2 demonstrating exceptional photosensitivity. The coordinated pattern of ZjHMGR1 and ZjHMGR3 expression suggests complementary roles in photomorphogenic recovery (Fig 7F and 7G). To minimize bias from a single accession, we conducted identical expression pattern analyses in an additional wild jujube accession (ZH#2). Results demonstrated that ZjHMGR1/2/3 maintained high root expression, while ZjHMGR2 exhibited constitutively low expression in stems, leaves, and flowers. ZjHMGR1/3 displayed ubiquitous expression across tissues—broadly consistent with Fig 7A. Following hormone and light treatments, ZjHMGR1/2/3 expression profiles in ZH#2 roots and leaves remained essentially identical to initial observations (S2 Fig in S1 File). The tripartite regulatory module implies that ZjHMGR1 as a candidate mediating root photomorphogenesis, ZjHMGR3 potentially involved in light responses and ZjHMGR2 serving as the master switch through its hyperresponsive light-responsive elements.

### 3.6. Inhibitive effects of saline-alkaline on seedlings growth of wild jujube

The phytohormone abscisic acid (ABA) serves as a central regulator of ionic homeostasis and osmotic adjustment in plant salinity tolerance networks, primarily through orchestrated activation of SOS pathway components and stress-responsive transcription factors (bZIP/NAC families) [9,51]. The robust transcriptional responsiveness of ZjHMGR paralogs to ABA induction strongly implicates this gene family in mediating wild jujube’s adaptive reprogramming under saline-alkaline stress conditions (Fig 7B and 7C). To mechanistically dissect ZjHMGR functionality in saline-alkaline stress adaptation, we recorded the seed germination rates of two varieties of wild jujube (ZH#1 and ZH#2) and established a controlled salinity-alkalinity composite stress system using hydroponically cultivated *Z. jujuba* var. *spinosa* seedlings (ZH#1). Germination assays of ZH#1 and ZH#2 sour jujube seeds under 0–200 mM NaCl gradients revealed marked dose dependency. Relative to controls, 50 mM NaCl exerted no significant impact on germination rates. However, 100 mM NaCl reduced germination to 38%, while 200 mM NaCl further suppressed germination to approximately 25% after 15 days (S3 Fig in S1 File and S5 Table). At early phase (0–12 h post-treatment), Absence of visible morphological changes despite massive transcriptomic remodeling, with 12422 genes exhibiting ≥1-fold expression shifts (S4 Fig in S1 File).

**Fig 8:**
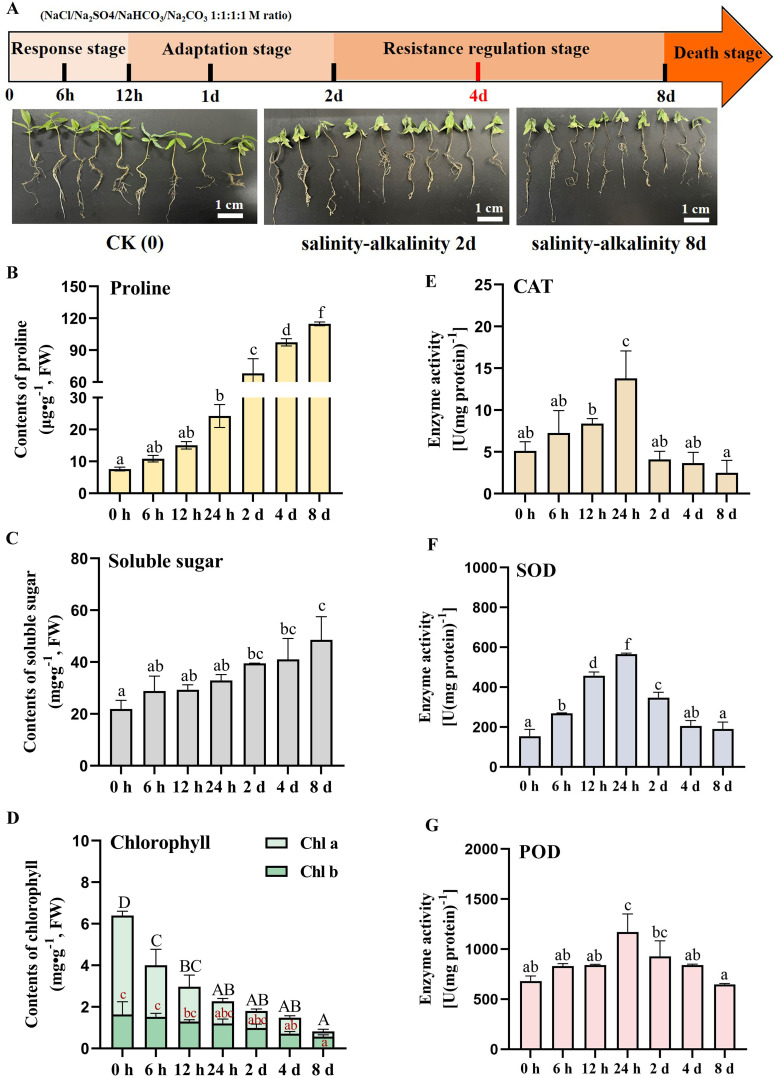
Analysis of phenotypic and physiological change patterns in *Ziziphus jujuba* var. *spinosa* seedlings under sustained salinity-alkalinity stress. (A) Phenotypes of wild jujube seedlings treated continuously with a saline-alkaline mixture of six parts per thousand. (B-D) The contents of proline (B), soluble sugar (C), and chlorophyll **(D)** of wild jujube seedlings treated continuously with a saline-alkaline mixture of six parts per thousand for 0-8 d. (E-G) The enzyme activity of CAT (E), SOD (F), and POD **(G)** of wild jujube seedlings treated continuously with a saline-alkaline mixture of six parts per thousand for 0-8 d. The saline-alkaline mixture of six parts per thousand consists of NaCl/Na_2_SO4/NaHCO_3_/Na_2_CO_3_ in the concentration ratio 1:1:1:1 M. Data are from three biological replicates (± SD). Statistical differences were shown by different letters according to One-Way ANOVA followed by Tukey’s HSD test, *P < 0.05, n = 10.

Integrated analysis of transcriptomic dynamics and phenotypic transitions enabled systematic classification of the composite stress response into four distinct adaptive satges: (1) response stag**e** (0–12 h), (2) adaptation stage (12 h-2 d), (3) resistance regulation stage (2–4 d), and (4) the death stage (>4 d) (Fig 8A). This multi-phase progression reflects an evolutionary-conserved adaption mechanism balancing energy allocation between stress perception and survival strategies. At response stage (0–12 h), the primary stress recognition window exhibited massive transcriptomic turbulence preceding visible symptomatology (S4 Fig in S1 File). Key biomarkers included photosynthetic collapse with significant reduction in chlorophyll a and ROS scavenging enzyme activation in superoxide dismutase (SOD) at this stage (Fig 8D and 8F). The salinity-alkalinity adaptation phase manifested between 12 h and 2 d post-treatment initiation (Fig 8A), characterized by sustained transcriptional activation of stress-responsive genetic elements (S4 Fig in S1 File). During this critical period, physiological analyses revealed three hallmark responses: (1) pronounced accumulation of osmolytes including proline and soluble sugars (Fig 8B and 8C); (2) progressive chlorophyll degradation (Fig 8D) and main root length shortened (S5 Fig in S1 File); and (3) transient activation of antioxidant defense systems. Notably, catalase (CAT), superoxide dismutase (SOD), and peroxidase (POD) activities exhibited stress-induced upregulation, peaking at 24 h (about 2.8-, 3.1-, and 2.1-fold increases, respectively) before subsequent attenuation (Fig 8E, 8F and 8G), indicative of dynamic redox homeostasis regulation. The resistance regulation phase (2–4 d) represented a pivotal transition in stress acclimatization. While phenotypic observations revealed partial seedling wilting (Fig 8A), molecular profiling demonstrated persistent upregulation of 5663 salinity-responsive genes (S4 Fig in S1 File), suggesting active transcriptional reprogramming in *Ziziphus jujuba* var. *spinosa*. This phase likely constitutes the functional window for salinity resistance gene networks in wild jujube, as evidenced by maintenance of delayed necrosis progression. Post 8-day exposure, seedlings entered the terminal stress phase marked by catastrophic physiological collapse: severe hydric deficit, severe chlorophyll depletion (Fig 8A and 8D), and massive leaf abscission. Meanwhile, osmolyte concentrations reached maximal levels (Fig 8B and 8C), and antioxidant enzyme activities plummeted to low levels (Fig 8E, 8F and 8G). Concurrent transcriptional profiling revealed aberrant gene activation patterns (S4 Fig in S1 File), suggesting that stress-induced senescence and death genes were highly upregulated. This phase was thus characterised as a state of death. These findings demonstrate that prolonged saline-alkaline exposure induces systemic dysfunction in wild jujube seedlings, disrupting the growth and cellular homeostasis through multi-level transcriptional and metabolic dysregulation.

### 3.7. Transcriptional reprogramming of MVA pathway genes under saline-alkali stress in wild jujube adaptation

**Fig 9:**
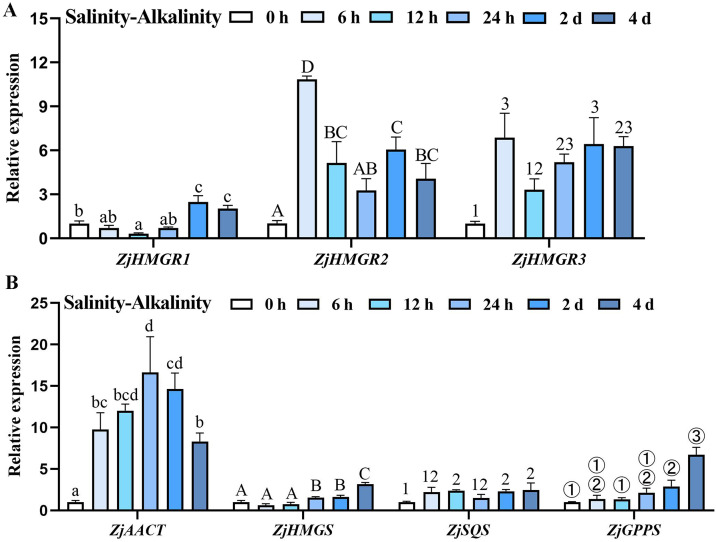
Expression analysis of MVA pathways-related genes in wild jujube seedings upon salinity-alkalinity treatment. (A-B) The transcription levels of *ZjHMGR* **(A)** and other MVA pathways-related genes **(B)** in four- to six-leaf stage seedings of wild jujube treated with a saline-alkaline mixture of six parts per thousand for 0-4 d. The relative gene expression was normalized to the level of the control and was normalized to *ZjACTIN7* expression. Data are from three biological replicates (± SD). Statistical differences were shown by different letters or numbers according to One-way ANOVA followed by Tukey’s HSD test, *P < 0.05.

To elucidate the regulatory interplay between *ZjHMGR* paralogs and saline-alkaline stress adaptation, we conducted temporal expression profiling of the *ZjHMGR* gene family across saline-alkali treatment gradients. qRT-PCR analysis revealed that all ZjHMGR isoforms exhibited significant stress induction (Fig 9A), with ZjHMGR2/3 displaying coordinated induction. These paralogs (ZjHMGR2 and ZjHMGR3) demonstrated rapid transcriptional activation, achieving peak expression at 6 h post-treatment followed by progressive attenuation (Fig 9A). Intriguingly, sustained stress exposure (>2 d) triggered a biphasic expression pattern, where transcript levels remained elevated relative to control despite post-peak decline, suggesting persistent metabolic demand for isoprenoid precursors under chronic stress. Intriguingly, comparative expression revealed functional divergence among *ZjHMGR* paralogs. While *ZjHMGR2/3* exhibited rapid stress responsiveness (6 h peak), ZjHMGR1 displayed delayed transcriptional activation, initiating significant upregulation only after 2 d sustained saline-alkali exposure (Fig 9A). This temporal specialization suggests stage-specific roles in ZjHMGR paralogs. ZjHMGR2/3 likely mediate acute saline-alkali adjustment, whereas ZjHMGR1 may orchestrate chronic stress adaptation. Mechanistically, the lack of co-expression correlation between ZjHMGR1 and ZjHMGR2/3 implies distinct regulatory architectures, potentially involving lineage-specific cis-regulatory elements or differential transcription factor binding. Collectively, these findings position the ZjHMGR family as central regulatory nodes in wild jujube’s salinity resistance network. The observed paralog-specific induction patterns and ZjHMGR1 independent regulatory decoupling exemplify evolutionary innovation in stress-responsive metabolic channeling.

As the key rate-limiting enzymatic step governing metabolic flux through the MVA pathway in *Ziziphus jujuba* var. *spinosa*, the ZjHMGR gene family may emerge as a critical regulatory node coordinating triterpenoid saponin biosynthesis and salinity adaptation. To dissect whether this evolutionarily conserved pathway exhibits stress-responsive rewiring, we performed time-resolved transcription profiling of core MVA pathway upstream enzymes – acetyl-CoA acyltransferases (*AACTs*) and 3-Hydroxy-3-methylglutaryl coenzyme A synthase (*HMGS*), and downstream of HMGR in the MVA pathway, squalene synthase (*SQS*) and geranylpyrophosphate synthetase (*GPPS*). The initial enzymatic component of MVA biosynthetic pathway, AACT, positioned upstream of HMGR, demonstrated immediate transcriptional activation under saline-alkaline stress conditions, catalyzing the condensation of acetyl-CoA to acetoacetyl-CoA (Fig 9B). In contrast, HMGS functioning upstream in the MVA pathway, exhibited delayed upregulation with attenuated expression magnitude relative to AACT under identical stress condition. Our analysis extended to critical downstream enzymatic targets – SQS and GPPS – both essential for pivotal triterpenoid saponin precursor. Quantitative analysis revealed distinct temporal expression patterns, SQS displayed rapid transcriptional induction within 12 h post-stress initiation, whereas GPPS mirrored HMGS’s delayed response profile, achieving significant upregulation only after sustained 24 h saline exposure (Fig 9B). The close relationship between activation of the MVA pathway from AACT to SQS/GPPS and stress response suggests that MVA-derived specialized metabolites, especially triterpenoid saponins, play the role of monitors during salinity stress tolerance in *Ziziphus jujuba* var. *spinosa*. These coordinated transcriptional dynamics suggest that HMGR serving as the central regulatory node of MVA pathway components in *Ziziphus jujuba* var. *spinosa* exhibit differential sensitivity to saline-alkaline stressors. In ZH#2 wild jujube seedings, MVA pathway-associated genes also exhibited time-dependent regulation under NaCl exposure (S6 Fig in S1 File). The observed transcriptional reprogramming provides evidence linking triterpenoid saponin biosynthesis with plant adaptation to saline-alkaline edaphic conditions by ZjHMGR.

### 3.8. Heterologous Overexpression of *ZjHMGR* Confers Enhanced Salinity Tolerance in *Arabidopsis*

**Fig 10:**
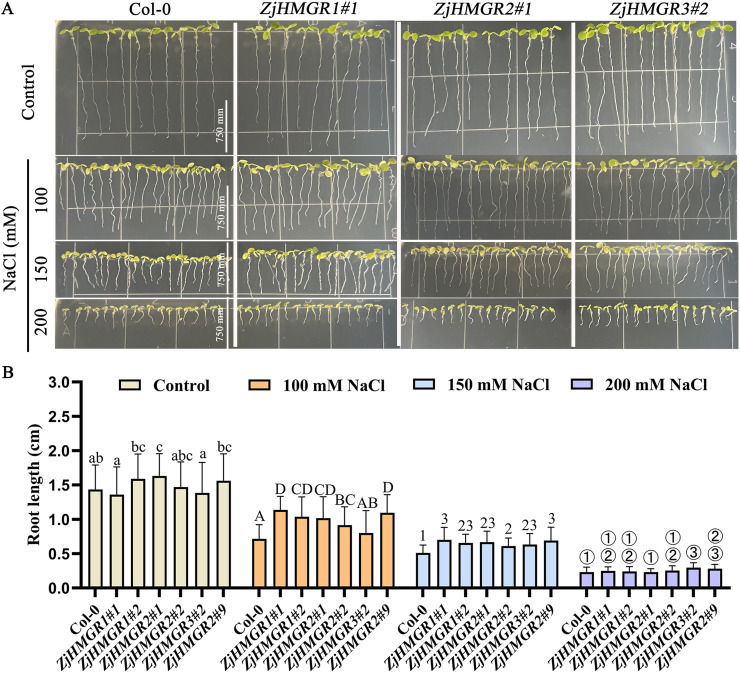
*A. thaliana* primary root growth during a 6-day period for the Col-0, *ZjHMGR1, ZjHMGR2* and *ZjHMGR3.* **(A)** Growth phenotypes of *A. thaliana* grown for 6 days in a vertically placed 1/2 MS medium. **(B)** Primary root length between Col-0 and *ZjHMGRs* transgenic lines. Data are from biological replicates (± SD). Statistical differences were shown by different letters or numbers according to One-Way ANOVA followed by Tukey’s HSD test, *P < 0.05, n ≥ 40 roots per overexpression strain.

Functionally validating ZjHMGR as a central regulator of the MVA pathway under saline-alkaline stress, we generated Arabidopsis lines overexpressing *ZjHMGR1*, *ZjHMGR2*, or *ZjHMGR3* (S7 and S8 Figs in S1 File). Primary root elongation assays revealed that while transgenic seedlings exhibited root lengths comparable to wild-type (Col-0) under control conditions, they displayed significantly enhanced tolerance under NaCl stress. Following 6-day exposure to 100 mM or 150 mM NaCl, root elongation in Col-0 was drastically attenuated. In contrast, all ZjHMGR-overexpressing lines maintained significantly longer root (Fig 10). Quantitative analysis showed a root length inhibition rate of 50.16% in Col-0 under 100 mM NaCl, markedly higher than the inhibition observed in ZjHMGR1 (16.32–34.77%), ZjHMGR2 (37.58–37.72%), and ZjHMGR3 (29.97–42.16%) transformants (S9 Fig in S1 File). A similar protective effect was evident at 150 mM NaCl, demonstrating a concentration-dependent response. Notably, at the severe stress level of 200 mM NaCl, *ZjHMGR3*-overexpressing lines uniquely sustained significantly longer roots compared to Col-0 and the other transgenics, where root lengths were comparable to wild-type, likely due to overwhelming ionic toxicity. Critically, *ZjHMGR* overexpression also enhanced shoot resilience. Transgenic lines exhibited increased biomass under non-stress conditions and maintained significantly higher fresh weight than Col-0 across all NaCl concentrations (100–200 mM; S10 Fig in S1 File). Collectively, these results demonstrate that *ZjHMGR* acts as a genetic determinant of salinity adaptation, conferring significant enhancement of whole-plant stress tolerance in *Arabidopsis*.

## 4. Discussion

Plants are exposed to numerous environmental changes throughout their life cycle, and the harshness of the survival environment makes cells unusually sensitive to stress signals and stimuli. Plant cells induce stress under a variety of adversity conditions such as heat, salinity, and drought. Salinity stress has become a type of abiotic stress that is in crisis globally and has a significant impact on plant survival. Soil salinization constitutes a pressing global ecological challenge that severely compromises agricultural sustainability by impairing plant growth trajectories, diminishing land productivity, and threatening food security networks. Nevertheless, China confronts multifaceted germplasm limitations in saline-alkaline land rehabilitation, characterized by three interdependent constraints: extensive spatial distribution of affected areas, paucity of halophytic cultivars with agronomic value, and suboptimal salt-tolerance thresholds in conventional crops. Recent climatic anomalies have precipitated a critical ecological transition in Chinese saline-alkaline ecosystems, with soil salt contents escalating to 6 ‰. This salt toxicity threshold now exceeds the adaptive capacity of most of commercial cultivars under controlled salinity trials. The strategic development of stress-resilient cultivars through advanced breeding technologies and germplasm innovation has emerged as a pivotal approach for maximizing saline soil productivity.

*Ziziphus jujuba* var. *spinosa* (wild jujube) emerges as a multifunctional phytoresource with potential encompassing pharmacotherapeutic, nutraceutical, ecological resilience, and agroeconomic dimensions [4,6]. Kernels of wild jujube are enriched with bioactive specialized metabolites, notably jujuboside A/B (triterpenoid saponins) and flavonoids, which mechanistically demonstrate superior anxiolytic efficacy, establishing this botanical as a first-line phytotherapeutic agent for neuropsychiatric disorders in traditional Chinese medical treatment [1,4,52]. This xerophytic perennial exhibits exceptional edaphic adaptability, demonstrating halophytic tolerance and drought resistance, but the molecular determinants underlying its extremophytic adaptation remain enigmatic. Jujuboside A has been identified as the principal bioactive constituent in *Ziziphus jujuba* var. *spinosa* seeds [1,4]. However, the biosynthetic confinement of these triterpenoid saponins presents three critical production bottlenecks, limited biomass yield exacerbated by climatic volatility, structural complexity and high price. Within the paradigm of low-abundance, high-value specialized metabolite engineering, this investigation proposes a synergistic strategy integrating mechanistic studies on the nexus between triterpene saponin biosynthesis in sour jujube and its halophytic adaptation. The methodology encompasses: (1) genome-wide identification and functional validation of rate-limiting enzymes within the mevalonate pathway (triterpenoid backbone biosynthesis) via multi-omics triangulation; (2) structure-activity relationship analyses coupled with molecular simulations to anticipate catalytic mechanisms and stress-responsive regulatory networks; and (3) establishment of high-fidelity homologous/heterologous expression chassis systems, thereby enabling secondary metabolism and adaptation to stress conditions. Such fundamental investigations will provide critical molecular insights for technological innovation in phytochemical production, efficient utilization of saline resources and ecological agriculture development.

### 4.1. Structural and Functional Conservation of HMGR: A Rate-Limiting Enzyme Governing Mevalonate Pathway Evolution in *Ziziphus jujuba* var. *spinosa*

Jujuboside triterpenoids, classified as dammarane-type tetracyclic compounds, are biosynthesized through the mevalonate (MVA) pathway, with 3-hydroxy-3-methylglutaryl-CoA reductase (HMGR) serving as the pivotal rate-limiting enzyme [4,25]. Contemporary molecular investigations demonstrate that HMGR gene characterization and functional elucidation significantly enhance triterpenoid saponin and terpenoid secondary metabolite yields in medicinal taxa including *Salvia miltiorrhiza* [29], *Panax ginseng* [28], *Tripterygium wilfordii* [53], and *Artemisia annua* [54]. Coordinated upregulation of mevalonate (MVA) pathway genes concurrently validates HMGR’s phylogenetically conserved regulatory mechanism in triterpenoid biosynthesis across divergent lineages. Despite *Ziziphus jujuba* var. *spinosa*’s medicinal prominence, its HMGR-mediated triterpenoid regulation remains underexplored. Systematic characterization of *ZjHMGR* isoforms, including structural phylogenetics and interactome mapping, will decode evolutionary function on metabolic networks for precision engineering of jujuboside biosynthesis.

In this paper, three members of the *ZjHMGR* gene family were identified in the wild jujube genome. To resolve potential genome annotation discrepancies, we performed orthogonal validation through integration of strand-specific RNA-seq data with molecular cloning followed by Sanger sequencing. This multimodal approach uncovered a critical misannotation in the original genome assembly, particularly in the exon-intron architecture of ZjHMGR1. The revised gene models, featuring corrected exon boundaries and phase consistency, have been deposited in the Supplementary Materials (S1 Table).*ZjHMGR1* and *ZjHMGR2*, which have similar gene structures, have similar protein physicochemical properties, whereas *ZjHMGR3*, which has lost one intron, resulting in a compact genomic architecture despite encoding the longest CDS (1,812 bp), has a large difference in protein physicochemical properties (Tables 1 and 2).ZjHMGR3’s intron loss correlates with distinct biophysical properties—acidic pI, lower instability index, and reduced α-helix content—suggesting structural stabilization (Tables 2 and 3). This may facilitate rapid transcriptional induction under saline stress (Fig. 9A), positioning ZjHMGR3 as a primary responder. All isoforms retained two catalytic signatures, HMG-CoA-binding motifs (EMPVGYVQIP, TTEGCLVA) and NADP(H)-binding domains (DAMGMNM, GTVGGGGT), confirming structural conservation (Fig 1 and 2). AlphaFold3 predictions (Fig 3) confirm all isoforms retain the conserved tripartite domain (N-terminal anchor, catalytic L-domain, S-domain). However, ZjHMGR3’s atypical C-terminal amphipathic helix may alter protein-protein interactions or subcellular dynamics. ZjHMGR protein structure and interspecific evolutionary relationships are highly conserved (Fig 3 and 4). Genome-wide covariate analysis detected two segmentally duplicated *ZjHMGR* pairs localized to distinct chromosomes (Fig 5A). The ancient origin (≈83 Mya) and persistent purifying selection highlight HMGR’s evolutionary stability as a rate-limiting enzyme in isoprenoid biosynthesis (Table 4). The duplication chronology further positions ZjHMGR1 as the ancestral lineage predating monocot–eudicot divergence, consistent with its collinearity to monocot HMGRs (Fig 5B). These dynamics exemplify how essential metabolic genes maintain functional fidelity despite genomic upheaval, ensuring terpenoid-mediated stress adaptation in extremophytes like *Z. jujuba* var. *spinosa*. Despite high sequence identity (>90% in catalytic domains), the differential intron architecture of ZjHMGR3 (loss of one intron; Table 1) suggests subfunctionalization. However, the minimal Ka/Ks values reject neofunctionalization, implying that divergent expression patterns (e.g., tissue specificity; Fig 7) arose via cis-regulatory evolution rather than coding-sequence changes. Thus, intron loss likely fine-tunes expression kinetics (e.g., rapid stress induction) rather than enzymatic function. Conserved synteny with stress-adapted species (*P. trichocarpa*, *V. vinifera*) implies selective maintenance of HMGR-mediated isoprenoid biosynthesis for abiotic stress adaptation in perennial woody plants.

Cis-regulatory element architecture in promoter regions critically governs spatiotemporal gene regulation. Photoresponsive HMGR expression modulation is evolutionarily conserved, evidenced by dark-inducible *HMGR1* upregulation in *Arabidopsis thaliana* immature leaves and light-repressed paclitaxel biosynthesis in fungal systems [55,56]. Promoter analysis of *Ziziphus jujuba* var. *spinosa* HMGR isoforms revealed abundant light-responsive cis-elements (Fig 6), corroborated by qRT-PCR demonstrating photoperiod-dependent regulation: leaf-specific induction versus root suppression (Fig 7). This tissue-divergent expression pattern suggests a novel regulatory paradigm where light modulates HMGR-mediated triterpenoid biosynthesis through isoform-specific transcriptional control, potentially driving metabolic regulation in medicinal plants. Promoter architecture analysis revealed *ZjHMGR* isoforms harbor enriched cis-regulatory modules, including phytohormone-responsive elements (ABRE, CGTCA/TGACG motifs), defense/stress-related motifs (TC-rich repeats, MYBHv1, CCAAT-box), and develomental indicators, suggesting HMGR’s integration into ABA-light-oxygen signaling networks (Fig 6). Acute *ZjHMGR* responsiveness, with pronounced transcriptional activation observed following ABA, MeJA, and salinity treatments were analyzed by qRT-PCR (Fig 7 and S2 Fig in S1 File). Specifically, ZjHMGR2 may serve as a primary responder to acute stress signals via its rapid ABA activation, and ZjHMGR3 potentially mediates prolonged adaptive responses through high-level induction. ZjHMGR3 displays unique spatiotemporal regulation—e.g., sustained ABA induction in roots but transient leaf response (Fig 7B and 7C). Its promoter harbors fewer MeJA-responsive elements, correlating with delayed MeJA response (24 h; Fig 7D and 7E). Given HMGR’s rate-limiting role in isoprenoid biosynthesis and ABA’s centrality in tripartite stress (salt/drought/osmotic) adaptation, we propose an evolutionary model where ZjHMGR isoforms functionally bridge specialized metabolism with systemic acquired resilience through terpenoid-hormone crosstalk. This multiple induction pattern suggests a complicated regulatory mechanism coordinating HMGR-mediated isoprenoid biosynthesis with environmental adaptation and stress resilience.

### 4.2. Elucidating the Regulatory Role of HMGR in Salinity Stress Adaptation of *Ziziphus jujuba* var. *spinosa*

*Ziziphus jujuba* var. *spinosa* exhibits sustained growth vigor in soils with high salinity, establishing its pioneer species status for phytoremediation in coastal salt marshes and secondary salinized inland ecosystems through physiological homeostasis regulation [57]. Under saline-alkaline stress, this adaptive mechanism not only mitigates ionic toxicity but also enables osmotic adjustment through compatible solute biosynthesis. Critical knowledge gaps persist regarding the temporal dynamics of stress-responsive phytohormones (e.g., ABA, JA) and the transcriptional regulatory cascades governing HMGR-mediated isoprenoid biosynthesis activation in this species. Integrative phenotyping, physiological profiling, and transcriptomic dissection revealed four sequential stress-response phases in *Ziziphus jujuba var. spinosa* seedlings under combined salinity-alkalinity stress: (1) response stag**e**, (2) adaptation stage, (3) resistance regulation stage, and (4) the death stage. Stage-specific phenotypic signatures and transcriptional reprogramming patterns were systematically characterized, with seedlings at the 4–6 true leaf stage demonstrating remarkable survival capacity (≥8 days) under 6‰ saline-alkaline co-stress (Fig 8, S4 and S5 Figs in S1 File). Combined salinity-alkalinity stress triggered multilevel physiological disruptions in wild jujube seedlings, manifesting as growth inhibition, reduced chlorophyll content, and osmotic accumulation. The combined salinity-alkalinity stress elicited a dual-phase osmotic response, marked by progressive proline accrual and soluble sugar elevation, consistent with osmolyte biosynthesis activation (Fig 8B and 8C). Paradoxically, this co-stress triggered temporal dysregulation of ROS homeostasis, while superoxide dismutase (SOD), peroxidase (POD), and catalase (CAT) activities exhibited transient enhancement (peaking at 24 h), systemic antioxidant capacity declined precipitously beyond this threshold, culminating in a redox collapse (Fig 8E,8F and 8G).As the central regulatory hub of plant stress adaptation, the endoplasmic reticulum (ER) orchestrates sophisticated cellular transcriptional reprogramming through activation of the unfolded protein folding and enhancement of co-translational processing, dynamic membrane lipid trafficking and remodeling [7]. This organelle integrates environmental sensing through phytohormone-mediated crosstalk with plasma membrane-localized receptors, triggering stress signaling cascades that modulate metabolic flux redistribution to maintain cell homeostasis [51].

Abscisic acid (ABA) exerts central regulatory control over plant salinity-alkalinity adaptation through coordinated phytohormonal signaling. ABA accumulation triggers an ABA-dependent signaling cascade that (1) activates stress-responsive transcriptional networks (e.g., *RD29B*, *ABI5*), (2) induces stomatal closure via ionic modulation, and (3) enhances osmoprotectant biosynthesis, collectively mitigating ionic imbalance under co-stress conditions. Strikingly, the *ZjHMGR* gene family exhibited ABA hypersensitization (induction within 6 h post-treatment; Fig 7B and 7C), positioning it as an early signaling hub in wild jujube’s salinity tolerance. Tissue-specific profiling revealed constitutive expression of *ZjHMGR1* and *ZjHMGR3* across organs (Fig 7A), with *ZjHMGR3*’s intronless architecture potentially enhancing transcriptional efficiency under acute stress (faster induction kinetics vs intron-containing paralogs; Fig 9A and S6A Fig in S1 File).Crucially, salinity stress coordinately upregulated the mevalonate (MVA) pathway (Fig 9B and S6B Fig in S1 File), demonstrating dual potential functionality in synthesizing triterpenoid saponins (key osmoprotectants) and responding to salinity through intermediate metabolites modulation or phytohormone-mediated crosstalk. This metabolic rewiring suggests evolutionary selection pressure favoring MVA pathway plasticity in halophytic *Ziziphus jujuba* var. *spinosa*. Targeted screening of high-saponin germplasm with *ZjHMGRs* allelic variants may thus accelerate breeding of salinity-resilient cultivars.

Phytohormones are central regulators of plant developmental processes and environmental stress adaptation. These endogenous signaling molecules are synthesised primarily by endogenous metabolic pathways. The ABA-, JA- and GAs-mediated phytohormone pathways directly regulate the transcriptional network for the synthesis of medicinal plant triterpenoids [58–62]. This transcriptional activation may be mediated by enhanced interactions with specific transcription factors, in particular ABRE, MYB and MYC2, ultimately promoting triterpenoid accumulation. The ZjHMGRs’ promoters contain several hormone-responsive cis-regulatory elements. The use of exogenous hormones during ZjHMGR-mediated regulation of triterpenoid biosynthesis may disrupt the homeostasis of the endogenous phytohormone network, thereby altering plant metabolic physiology. However, the potential functions of HMGR, either as a regulatory link between phytohormone signaling and terpenoid accumulation or as a quantitative switch, remain unresolved due to the complexity of hormonal interactions, the different expression patterns of terpenoid synthase genes and the dynamic interactions between transcription factors and the promoter in this multi-faceted regulatory process.

To functionally characterize the *ZjHMGR* gene family in salinity adaptation, we engineered three binary expression vectors (*pROKII-35S::ZjHMGR1/2/3-eGFP*) (S7 Fig in S1 File) and generated stable transgenic *Arabidopsis* lines through *Agrobacterium*-mediated floral dip transformation (S8A Fig in S1 File). Antibiotic-based selection yielded a T1 transgenic population (HMGR1: n = 9; HMGR2: n = 3; HMGR3: n = 21, independent lines per construct) for subsequent functional investigations(S8B Fig in S1 File). Upon establishing homozygous T3 transgenic lines, qRT-PCR confirmed effective ZjHMGR overexpression, with rigorous validation ensuring reproducibility across replicates (S8C Fig in S1 File). Based on the robust phenotypic validation in heterologous systems, we demonstrate that *ZjHMGR1/2/3* overexpression confers enhanced salinity tolerance in *Arabidopsis*, significantly mitigating NaCl-induced root growth inhibition (16–42% vs. 50% in WT at 100 mM NaCl) and preserving shoot biomass (Fig 10, S9 and S10 Fig in S1 File). Crucially, *ZjHMGR3* uniquely maintained root elongation under extreme stress (200 mM NaCl), aligning with its distinct structural features (intron loss; Table 1) and rapid stress-induction kinetics in native jujube (Fig 9A). These findings functionally anchor *ZjHMGRs* as key genetic determinants of halophytic adaptation in *Z. jujuba* var. *spinosa*. Crucially, this dual-functional function enables multi-omics dissection of ZjHMGR-mediated triterpenoid saponin biosynthesis, providing a chassis for metabolic engineering, and development of an allele swapping platform where ZjHMGR can be functionally evaluated to identify salinity-resilient alleles for marker-assisted breeding. Moreover, these studies will be instrumental in formulating a strategy to enhance the resilience of wild jujube to unfavourable environmental conditions, thereby ensuring its survival and the development of superior and highly efficient anticlimactic wild jujube germplasm resources.

## Supporting information

S1 File Supplementary figures. (DOCX)

S1 Table Details of HMGR corrected sequences used in this study. (XLSX)

S2 Table and S3 Table Primers for quantitative real-time PCR and vector construction. (DOCX)

S4 Table Gene ID and Sequences of HMGR in evolution tree. (XLSX)

S5 Table Germination rates of two varieties of wild jujube under salt stress. (XLSX)
