# Supplementary material for: Genome-Wide identification and salt stress-responsive expression dynamics of the HMGR gene family in Ziziphus jujuba var. spinosa
Source: PLoS One. 2025 Aug 20;20(8):e0330439. doi: 10.1371/journal.pone.0330439 (PMC12367118; doi:10.1371/journal.pone.0330439)
Supplement: S1 File — (DOCX) [file pone.0330439.s001.docx]

**Supplementary figures**


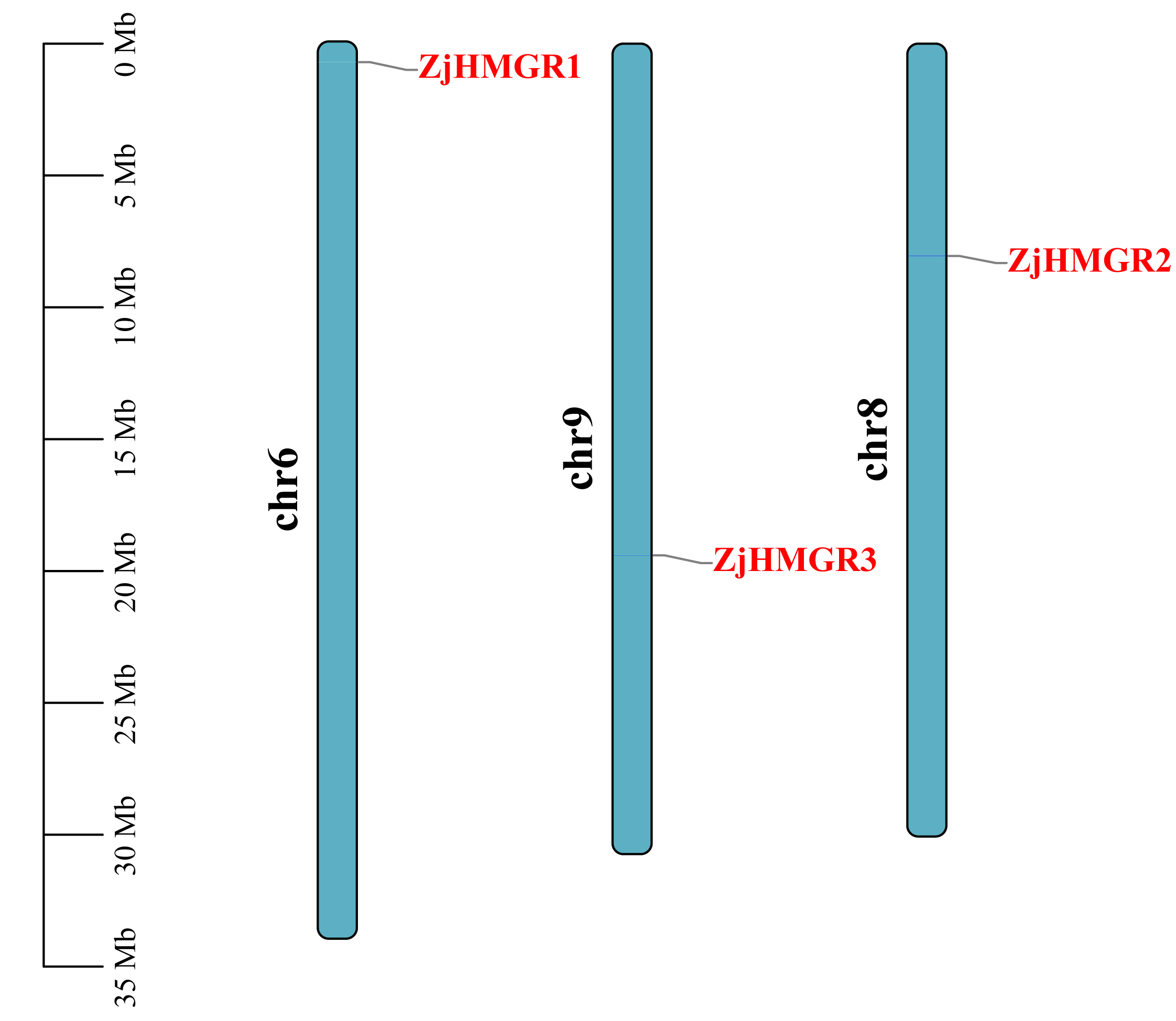


**Figure S1.** **Schematic modle of *HMGR* (*ZjHMGR*) genes distribution on *Ziziphus jujuba* var. *spinosa* three chromosomes.** A total of 3 *ZjHMGR* genes were mapped to three chromosomes. The number of chromosomal is found on the left of each chromosome (Chr), along with the gene’s name in red on the right side.


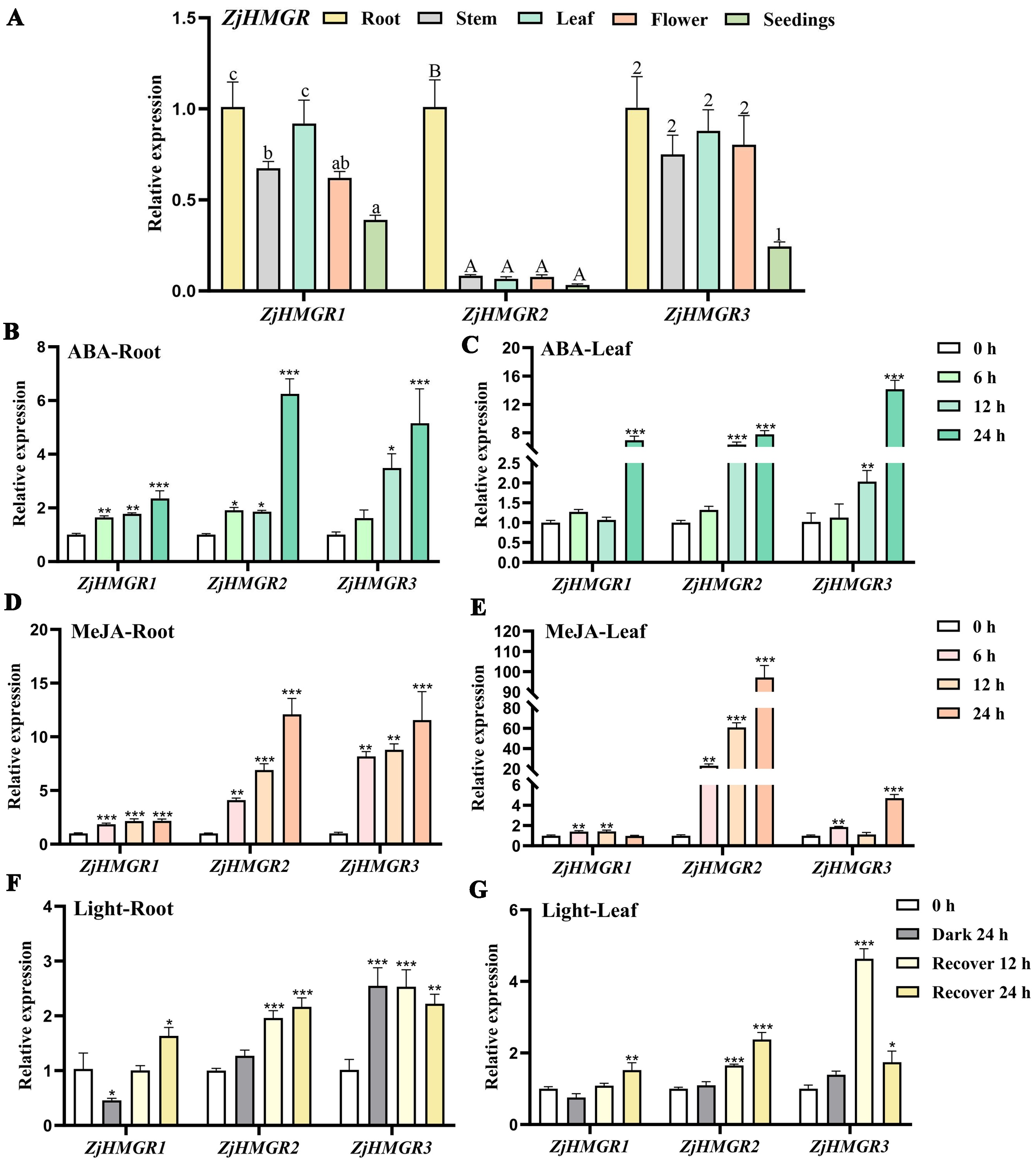


**Figure S2. Tissue-specific expression of *ZjHMGR* and analysis of expression patterns in ZH#2 upon different stresses and exogenous hormone treatments. (A)** The transcription level of *ZjHMGR* in different tissues of 5-year-old plants and four- to six-leaf stage seedings in ZH#2 assayed by qRT-PCR. **(B–E)** The transcription level of *ZjHMGR* in four- to six-leaf stage seedings of ZH#2 treated with exogenous ABA or MeJA assayed by qRT-PCR. The levels in roots or leaves of seedlings in the absence or presence of 100 μM ABA or 1 mM MeJA for 0-24 h. **(F-G)** The transcription level of *ZjHMGR* in four- to six-leaf stage seedings of ZH#2 protected from light for 24 h, and then recovered to light for 12 h or 24 h. The relative gene expression was normalized to the level of the control and was normalized to Zj*ACTIN7* expression. Data are from three biological replicates (± SD). Statistical differences were shown by different letters or asterisks according to One-Way ANOVA followed by Tukey's HSD test, **P* < 0.05, ***P* < 0.01, ****P* < 0.01.


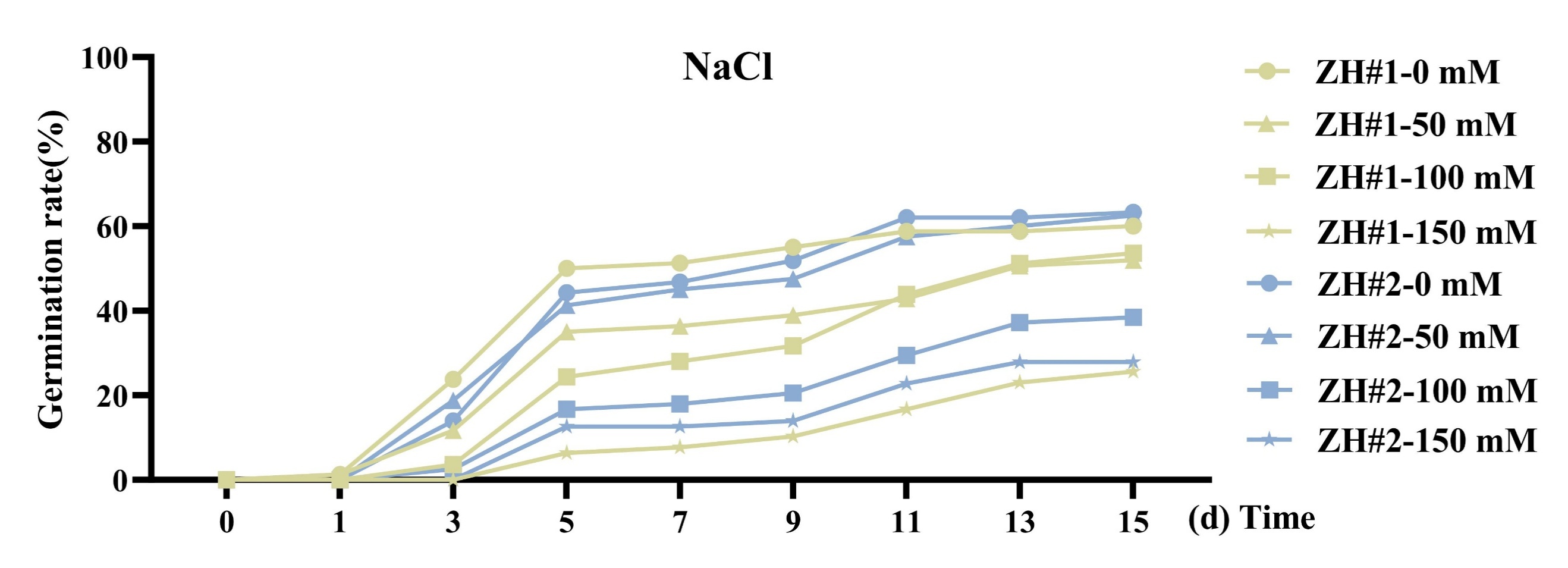


**Figure S3. The seed germination rates of two varieties of wild jujube (ZH#1 and ZH#2) were investigated.** Wild jujube seeds were separately placed on moist filter paper containing 0, 50, 100, or 150 mM NaCl solutions and cultivated continuously for a period of 15 days. Germination rates were recorded at two-day intervals. Detailed germination rate data can be found in Supplementary Table S5.


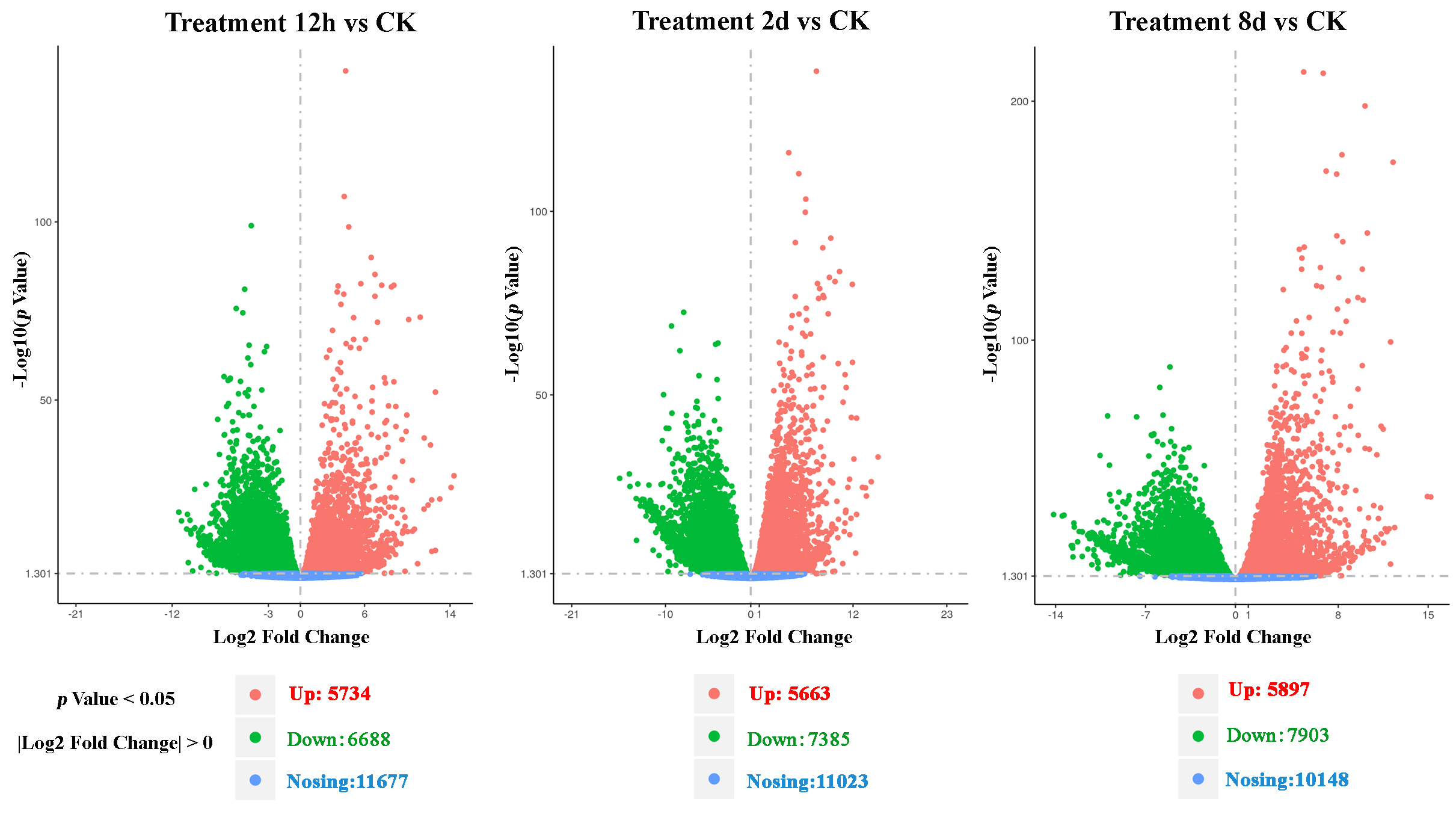


**Figure S4.** **RNA sequencing of *Ziziphus jujuba* var. *spinosa* seedings showed the effects on salinity-alkalinity stress resistance.** A plot of gene expression differences between control and treated groups of wild jujube seedlings with a saline-alkaline mixture of six parts per thousand for 12 h, 2 d or 8 d. Significantly up- or down-regulated genes are indicated by red or green circles,
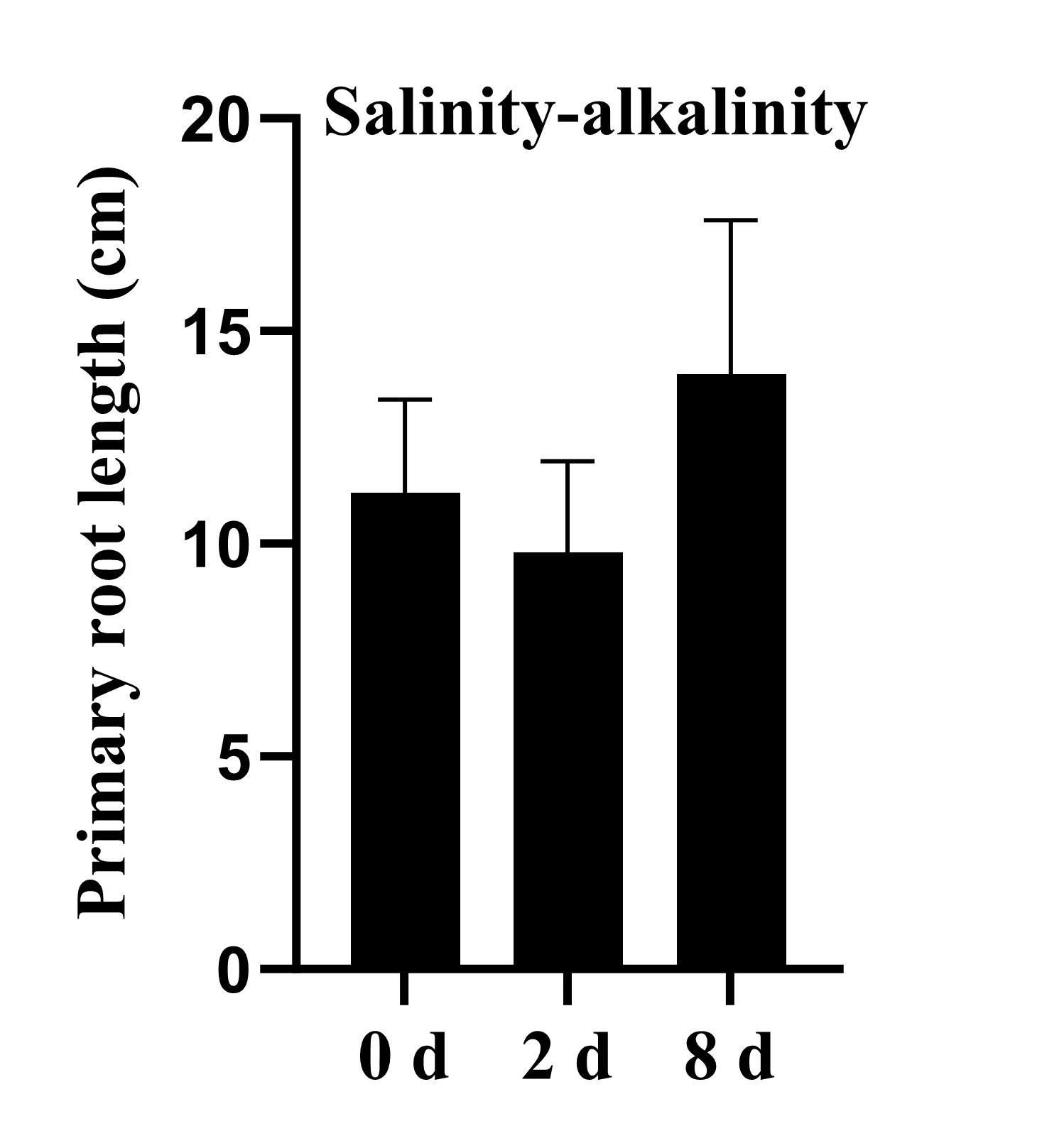
respectively, whilst other data points are plotted in blue.

**Figure S5. Analysis of main roots length in *Ziziphus jujuba* var. *spinosa* seedlings under sustained salinity-alkalinity stress.** Root length was measured by ImageJ. n ≥ 9.


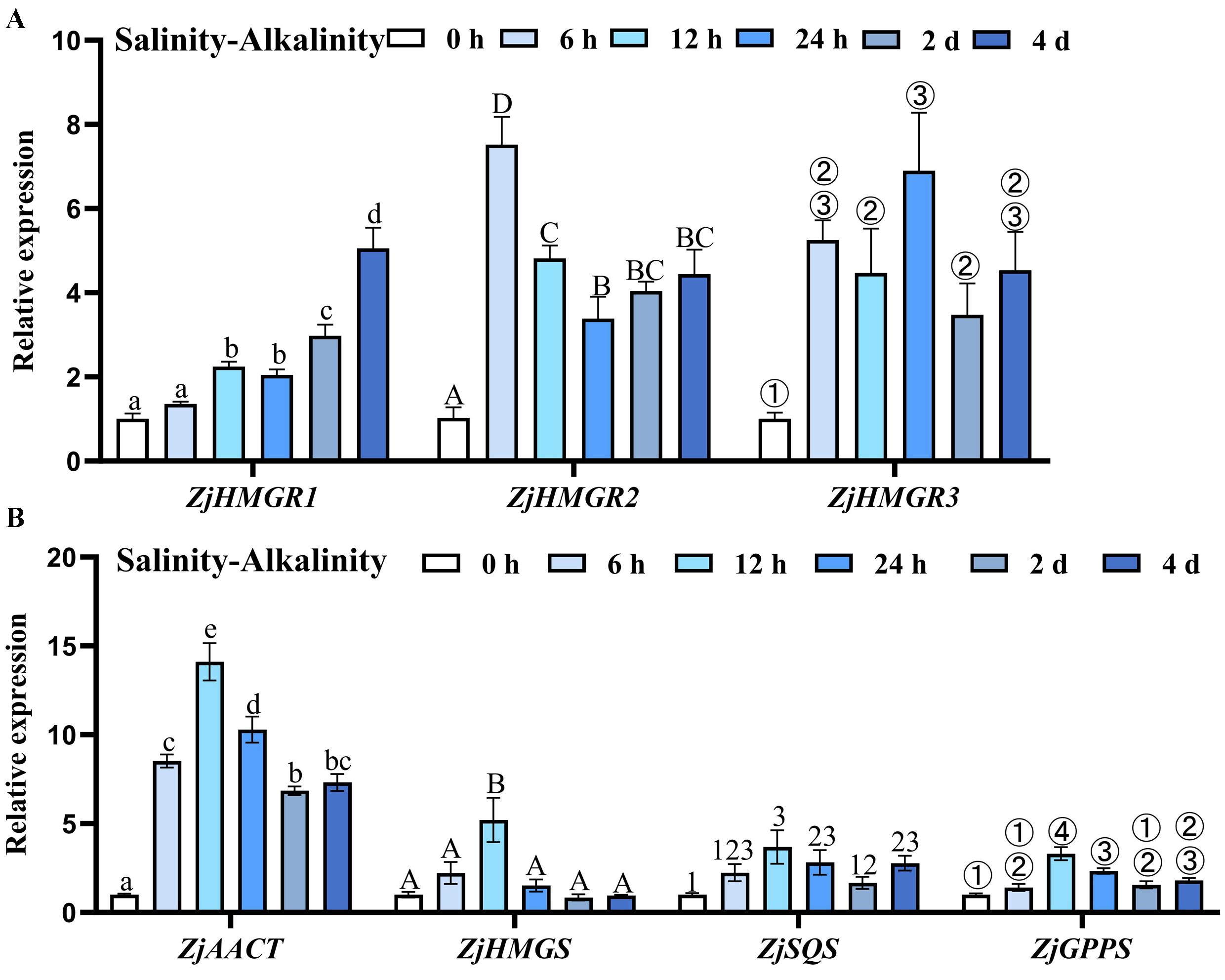


**Figure S6. Expression analysis of MVA pathways-related genes in wild jujube (ZH#2) seedings upon salinity-alkalinity treatment.** **(A-B**) The transcription levels of *ZjHMGR* **(A)** and other MVA pathways-related genes **(B)** in four- to six-leaf stage seedings of wild jujube (ZH#2) treated with a saline-alkaline mixture of six parts per thousand for 0-4 d. The relative gene expression was normalized to the level of the control and was normalized to *ZjACTIN7* expression. Data are from three biological replicates (± SD). Statistical differences were shown by different letters or numbers according to One-way ANOVA followed by Tukey's HSD test, **P* < 0.05.


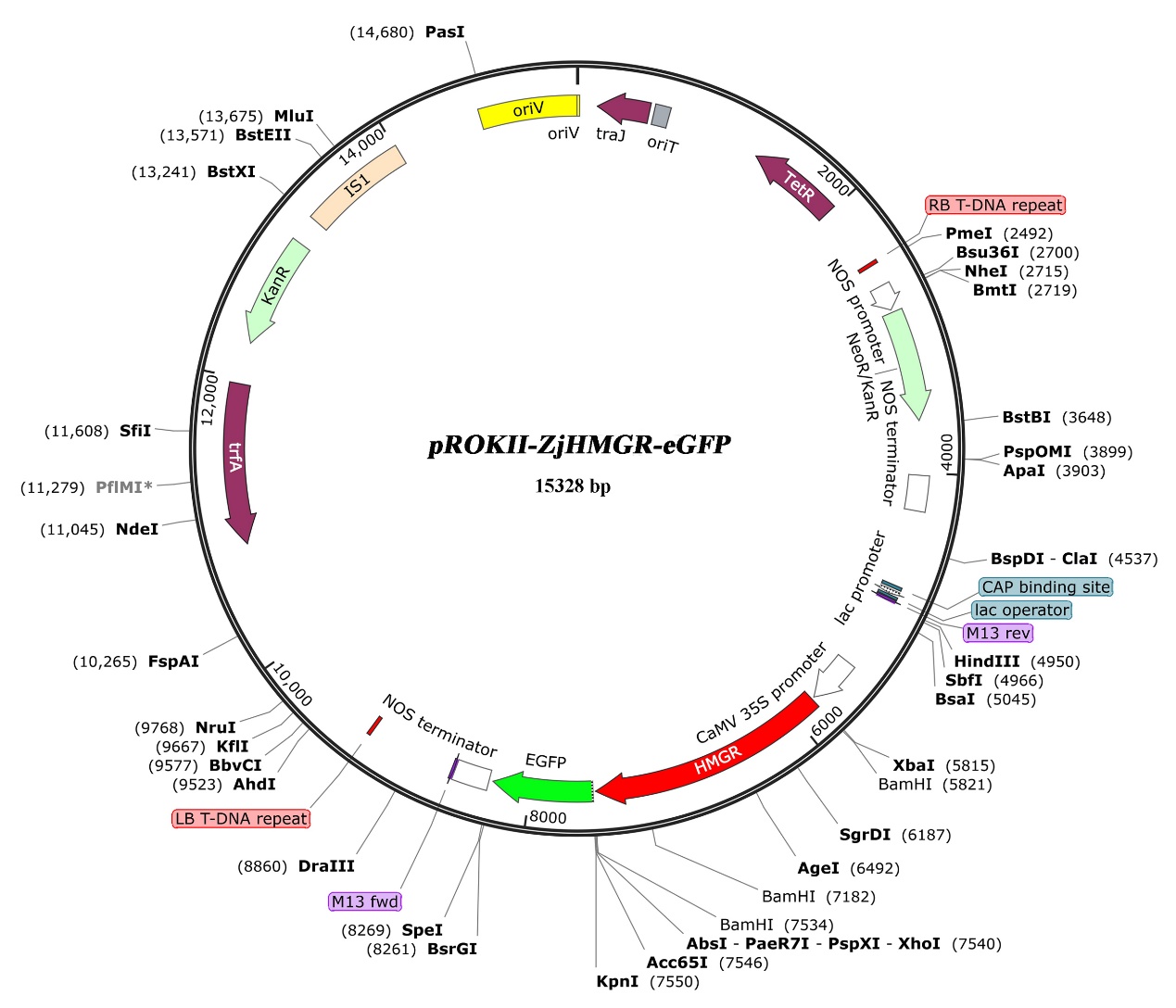


**Figure S7.** **Schematic diagrams of *35S:pROK2-ZjHMGR-eGFP* constructs.** Schematic representation of recombinant vector construct with genetic element annotation: kanamycin resistance selection marker (NeoR/KanR); HMGR insertion locus (red arrowhead); eGFP fluorescence reporter (green arrowhead).


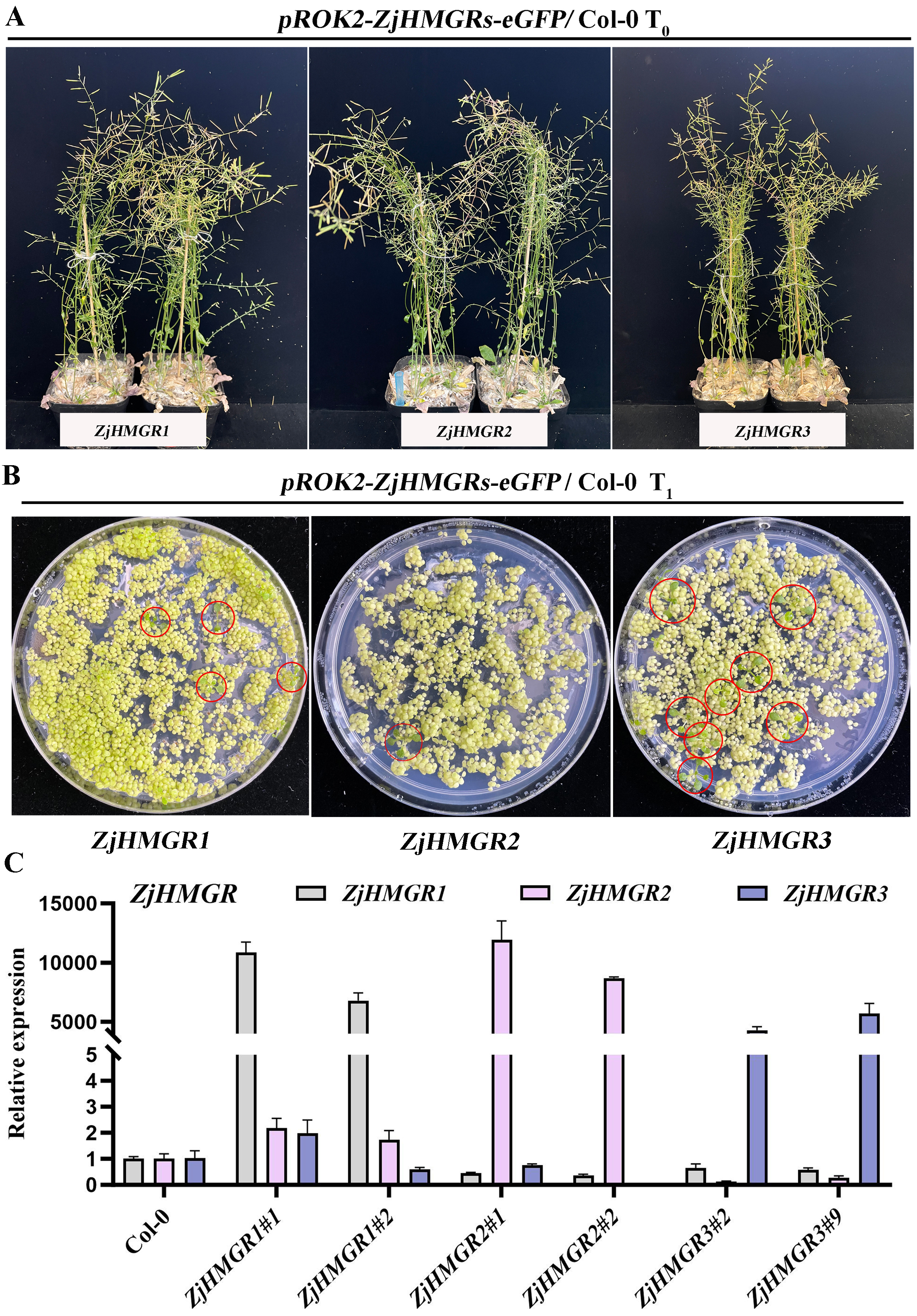


**Figure S8.** **Construction *ZjHMGR* overexpression heterologous transformants in *Arabidopsis thaliana.* (A)** *35S:pROK2-ZjHMGR-eGFP* constructs was heterologously transformed into *Arabidopsis* wild-type Col-0 to obtain *ZjHMGR* overexpression heterologous transformants for gene function studies. **(B)** Identification of *ZjHMGR*-overexpressing transgenic lines through kanamycin selection in T1 generation progeny, with positive transformants demarcated (red circles). **(C)** Identification of *ZjHMGR*-overexpressing transgenic lines by qRT-PCR in T3 generation progeny. The transcription level of *ZjHMGR* in 8-day-old *Arabidopsis thaliana* seedings. The relative gene expression was normalized to the level of the control and was normalized to At*ACTIN8* expression. Data are from three biological replicates (± SD).


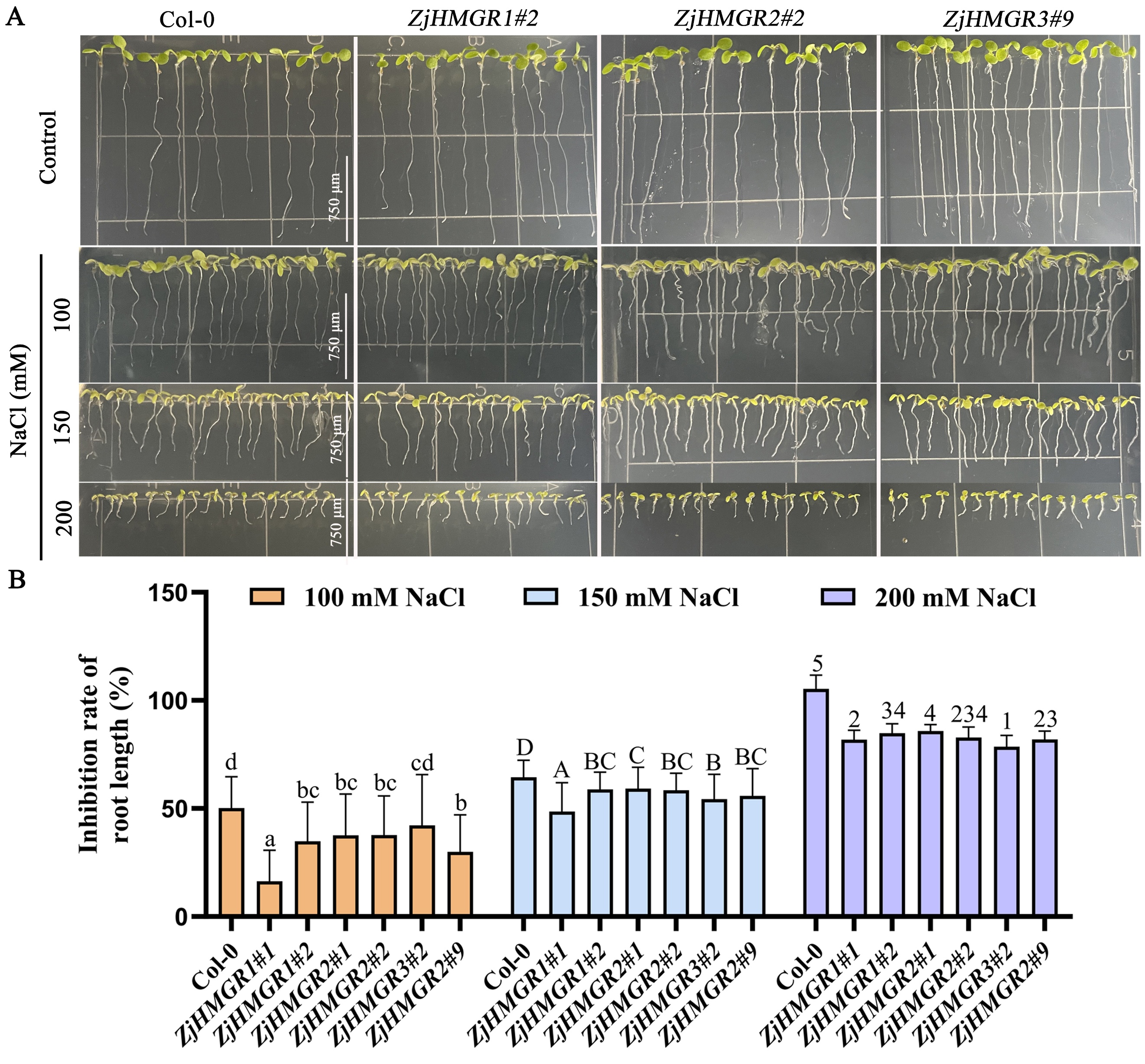


**Figure S9. Inhibition rate of root length in primary root growth of the Col-0, *ZjHMGR1,* *ZjHMGR2* and *ZjHMGR3*** **transgenic lines during a 6-day period in** *A. thaliana***. (A)** Root phenotypes of *A. thaliana* grown for 6 days in a vertically placed 1/2 MS medium with 0-200 mM NaCl. **(B)** Inhibition rate of primary root length between Col-0 and *ZjHMGRs* transgenic lines. Data are from biological replicates (± SD). Statistical differences were shown by different letters or numbers according to One-Way ANOVA followed by Tukey's HSD test, **P* < 0.05, n ≥ 40 roots per overexpression strain.


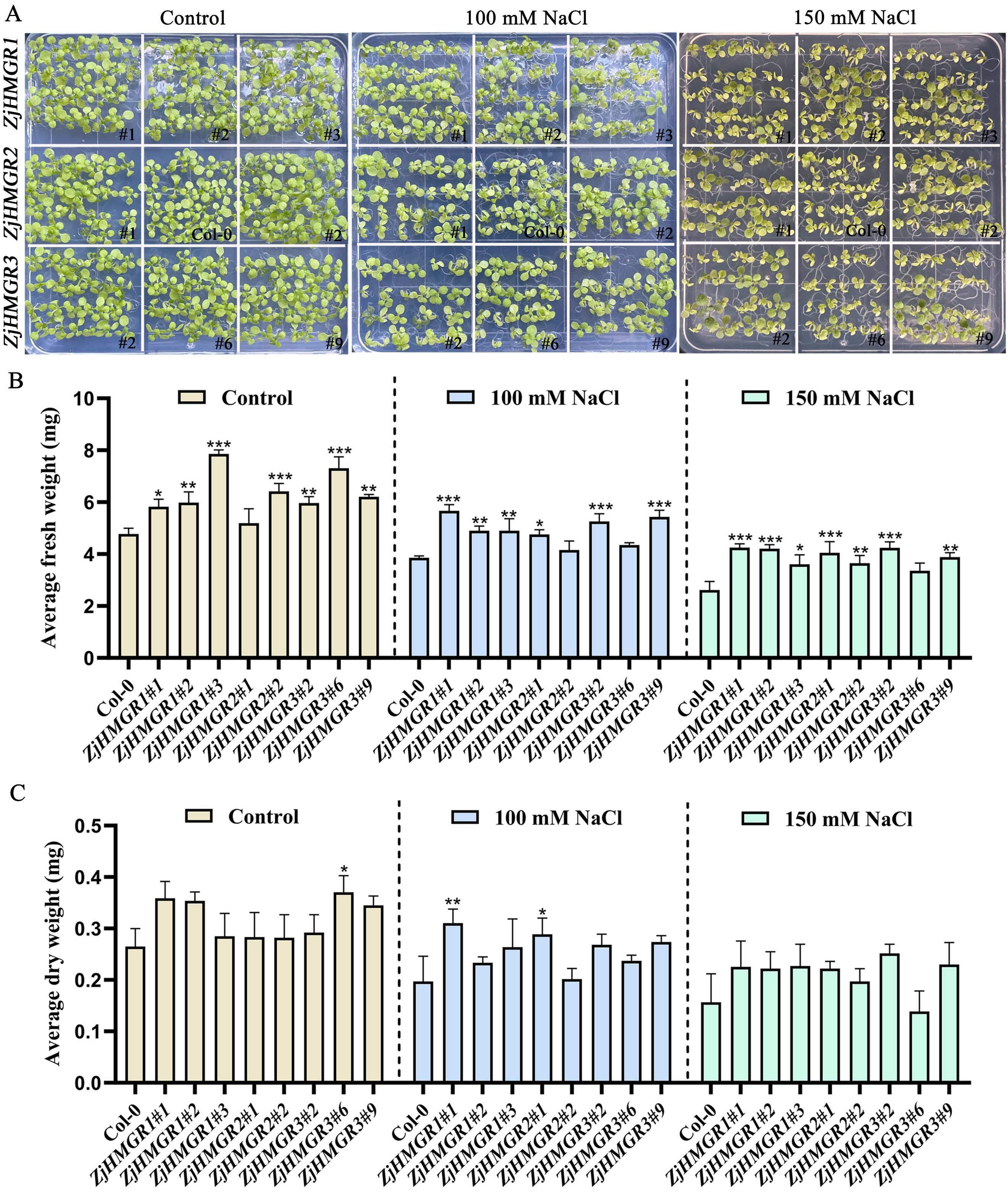


**Figure S10. *A. thaliana* shoot growth during a 8-day period for the Col-0, *ZjHMGR1,* *ZjHMGR2* and *ZjHMGR3*. (A)** Growth phenotypes of *A. thaliana* grown for 8 days in a 1/2 MS medium. **(B-C)** Fresh weight **(B)** and dry weight **(C)** between Col-0 and *ZjHMGRs* transgenic lines. Data are from three biological replicates (± SD). Statistical differences were shown by asterisks according to One-Way ANOVA followed by Tukey's HSD test, **P* < 0.05, ***P* < 0.01, ****P* < 0.001.
