# Supplementary material for: Genome-Wide identification and salt stress-responsive expression dynamics of the HMGR gene family in Ziziphus jujuba var. spinosa
Source: PLoS One. 2025 Aug 20;20(8):e0330439. doi: 10.1371/journal.pone.0330439 (PMC12367118; doi:10.1371/journal.pone.0330439)
Supplement: S2 Table and S3 Table — (DOCX) [file pone.0330439.s003.docx]

**Supplementary Table 2. Primers for quantitative real-time PCR**

| Primer name | Primer sequence (5'-3') |
| --- | --- |
| ZjACTIN7-qF | CTCAGCACCTTCCAGCAGATGT |
| ZjACTIN7-qR | CGACCCCACCTCAATGCTTC |
| ZjHMGR1-qF | ATGCCTTCAATTGAGGTTGGTA |
| ZjHMGR1-qR | GACAGTTCCCCAGCCAACA |
| ZjHMGR2-qF | TGCCTTCCATTGAGGTGGGC |
| ZjHMGR2-qR | GCTGCAAGAGCAGACATGAGA |
| ZjHMGR3-qF | ACCGCTAAAAGAGCTGCTGA |
| ZjHMGR3-qR | TCCCAAATCTGCTTGATCTGTTGA |
| ZjAACT-qF | TCTCGCAGAAGCAAGGAAGG |
| ZjAACT-qR | AAACTCCCATGCCAGCATCA |
| ZjHMGS-qF | ACAGACAGTGCGGTTTATGC |
| ZjHMGS-qR | ATTCACTTGCGAGGTCAGGC |
| ZjSQS-qF | ACCTCCGTAACGCTGTATGC |
| ZjSQS-qR | ACATCGGCAGGTATGCTTGT |
| ZjGPS-qF | CCGGGATTTCTTGCCTCCTT |
| ZjGPS-qR | CAAGCTTAGGAACCTCGGCA |
| AtACTIN8-qF | TCAGCACTTTCCAGCAGATG |
| AtACTIN8-qR | ATGCCTGGACCTGCTTCAT |

**Supplementary Table 3. Primers for vector construction**

| Primer name | Primer sequence (5'-3') |
| --- | --- |
| ZjHMGR1-F | acgggggactctagaggatccATGGATATCCGCCGGCGGCCGCCCA |
| ZjHMGR1-R | catggtaccctcgagggatccTGTTCCTTCACTTGCTGCTTTGGACACGTCCTTG |
| ZjHMGR2-F | acgggggactctagaggatccATGGACGTTCGCCGGCGAAATACCAAAC |
| ZjHMGR2-R | catggtaccctcgagggatccATTGGCCATTTTAGAGACATCCTTGCTAGATCTGTTA |
| ZjHMGR3-F | acgggggactctagaggatccATGGACGTTCGCAGGCGATC |
| ZjHMGR3-R | catggtaccctcgagggatccGGCGGAAGCAGCAGCAGTC |
